# Identification and comparison of key RNA interference machinery from western corn rootworm, fall armyworm, and southern green stink bug

**DOI:** 10.1371/journal.pone.0203160

**Published:** 2018-09-05

**Authors:** Courtney Davis-Vogel, Brandon Van Allen, John L. Van Hemert, Amit Sethi, Mark E. Nelson, Dipali G. Sashital

**Affiliations:** 1 Research and Development, DuPont Pioneer, Johnston, Iowa, United States of America; 2 Roy J. Carver Department of Biochemistry, Biophysics, and Molecular Biology, Iowa State University, Ames, Iowa, United States of America; University of Tennessee, UNITED STATES

## Abstract

RNA interference (RNAi)-based technology shows great potential for use in agriculture, particularly for management of costly insect pests. In the decade since the insecticidal effects of environmentally-introduced RNA were first reported, this treatment has been applied to several types of insect pests. Through the course of those efforts, it has become apparent that different insects exhibit a range of sensitivity to environmentally-introduced RNAs. The variation in responses across insect is not well-understood, with differences in the underlying RNAi mechanisms being one explanation. This study evaluates eight proteins among three agricultural pests whose responses to environmental RNAi are known to differ: western corn rootworm (*Diabrotica virgifera virgifera*), fall armyworm (*Spodoptera frugiperda*), and southern green stink bug (*Nezara viridula*). These proteins have been identified in various organisms as centrally involved in facilitating the microRNA- and small interfering-RNA-mediated interference responses. Various bioinformatics tools, as well as gene expression profiling, were used to identify and evaluate putative homologues for characteristics that may contribute to the differing responses of these insects, such as the absence of critical functional domains within expressed sequences, the absence of entire gene sequences, or unusually low or undetectable expression of critical genes. Though many similarities were observed, the number of isoforms and expression levels of double-stranded RNA-binding and argonaute proteins varied across insect. Differences among key RNAi machinery genes of these three pests may impact the function of their RNAi pathways, and therefore, their respective responses to exogenous RNAs.

## Introduction

Control of agricultural pests through use of genetically engineered crops is a critical aspect of the integrated approach needed to provide sustainable food supplies for a growing world population [1]. Utilization of RNA interference (RNAi)-based technology in genetically engineered crops is currently being widely explored for insect pest management [2]. RNAi encompasses three related mechanisms of control at the RNA level, wherein RNA is targeted for repression or degradation through the action of microRNA (miRNA), small interfering RNA (siRNA), or Piwi-interacting RNA (piRNA). This general process, first discovered in *Petunia* and described in *Caenorhabditis elegans* (*Cel*) [3, 4], is highly conserved across plants and animals. Hijacking the RNAi pathways mediated by miRNAs and siRNAs has been shown to be effective in controlling insect damage to crop plants [5, 6], though the RNAi pathways of these pests are not well-characterized. It is known from model systems that pathways within a single organism differ in the proteins involved, the source of precursor RNA from which small RNAs (sRNAs) are generated, and the exact mechanism and outcome of target RNA silencing (reviewed in [7, 8]). The RNAi pathways of *Drosophila melanogaster* (*Dme*) are among the best understood. Consequently, the *Dme* pathways may serve as an appropriate model system for study of RNAi in other insects, though it remains unclear how much mechanistic information may be reliably extrapolated. Many *Dme* proteins involved in RNAi have been identified, including several nucleases that produce sRNAs, their associating double-stranded RNA binding protein (dsRBP) partners, and argonaute proteins. As the components most directly involved in the interference response, these are considered “core” RNAi machinery and are the focus of the current study.

The miRNA pathway functions primarily in regulation of gene expression (reviewed in [9]), while the siRNA pathway is thought to be an ancient defense against invading viruses—a process especially critical for insects (reviewed in [9, 10]). At the start of both pathways, long single-stranded or double-stranded (ss- or ds-) RNA is enzymatically processed into small dsRNA duplexes of ~20–30 nucleotides (nt). RNAs that trigger the miRNA pathway are typically endogenously expressed and sources may include specially transcribed non-coding RNA, or pre- or post-excised introns. In contrast, the siRNA pathway is usually activated by long, perfectly base-paired exogenous dsRNAs. In *Dme*, single-stranded primary miRNAs are shortened to precursor miRNAs in the nucleus by the microprocessor complex, composed of Drosha and its partner dsRBP Pasha [11, 12]. After precursor miRNAs are exported from the nucleus to the cytoplasm, they follow a path similar to dsRNAs uptaken by *Dme* cells. Dicer-1 (DCR-1) associates with and processes precursor miRNAs into small ~22 nt duplexes [13], whereas long siRNA-activating dsRNAs associate with and are processed into 21–25 nt duplexes by Dicer-2 (DCR-2) [14].

One strand of a sRNA duplex generated by either pathway is loaded into an argonaute protein family member, usually assisted by a dsRBP, forming an RNA-induced silencing complex (RISC). The RISC binds complementary ssRNA targets, resulting in their silencing. A mature miRNA strand is loaded into Argonaute 1 (AGO1) by DCR-1 and the dsRBP Loquacious (LOQS) to form the miRNA RISC (miRISC) [15–19]. In an analogous process, a guide siRNA is preferentially loaded into Argonaute 2 (AGO2) with the assistance of DCR-2 and the dsRBP R2D2 to form the siRNA RISC (siRISC) [20–22]. The final specificities of RISCs for their targets generally differ from one another. A miRISC will bind nt segments—typically within the 3’ untranslated regions of transcripts—containing exact complementarity to the miRNA seed sequence, commonly positions 2–8 from the miRNA 5´ end (reviewed in [23]). The remainder of the miRNA sequence may contain imperfect complementarity, resulting in one miRNA having the ability to regulate many transcripts through translational repression. The active siRISC binds cellular ssRNA exhibiting high complementarity along the full length of the guide strand [24]. Once bound, such ssRNA is cleaved by AGO2; the 21–25 nt complementarity typically results in a one-to-one pairing of siRNA and target [24].

It is clear from the study of both model and non-model systems that basic RNAi mechanisms are present in all plants and animals. However, differences across organisms in the protein machinery involved can affect various characteristics of the response, including type of mediating sRNAs, life stages and tissues in which different pathways function, and overall regulatory outcomes [8, 10, 25]. More specific to the application of RNAi for insect control, response to environmentally-introduced dsRNA (environmental (e)RNAi) is known to vary greatly across species. Many insects from the order Coleoptera tend to show robust activation of their RNAi pathways via oral feeding on transgenic plants expressing dsRNAs, whereas even in a laboratory setting, dsRNA elicits little response in Lepidoptera or Hemiptera (reviewed in [26, 27]). A variety of factors have been proposed to contribute to these differences, such as disparity in dsRNA uptake mechanisms and nuclease content of saliva and gut fluid [26]. Much focus has been placed on these initial barriers to treatment with insecticidal dsRNA [28], though less is known about downstream factors which may also play a role.

Understanding differences in insect response to eRNAi is central to the development and proper implementation of RNAi-based crop protection. This need, along with a general lack of research in prominent agricultural pests, led to focus of the current study on the core RNAi machinery of *Diabrotica virgifera virgifera* (western corn rootworm–WCR), *Spodoptera frugiperda* (fall armyworm–FAW), and *Nezara viridula* (southern green stink bug–SGSB). These three representative pests were used to explore another possible source of variability in RNAi efficacy: differences in the presence or absence, modifications to, or expression levels of core RNAi pathway proteins [26, 27]. Reports describing predicted protein features and phylogeny of a handful of core RNAi machinery genes in WCR and FAW are available [29–32]. However, given the complexity revealed through decades-long study of *Dme* RNAi, more work is needed to understand the differences that might exist between core RNAi components of insects that respond well to control via eRNAi and those that do not.

This study is aimed at exploring differences in the core RNAi machinery of WCR, FAW, and SGSB. Toward that end, identification of core mi- and siRNA components in each insect was performed, followed by prediction and comparison of protein domain structure, phylogeny, and inspection of expression patterns across life stages. Furthermore, a direct comparison of the baseline expression levels across insects was conducted for each core component. While the putative homologues identified in these pests are similar to reference sequences and demonstrate some consistency across insect in expression patterns and levels, detailed examination reveals intriguing differences. The number, features, and expression of the dsRBP and AGO isoforms show variations that may influence basic functioning of the RNAi pathways in these insects.

## Results

### *In silico* identification of core RNAi machinery

Putative homologues of all eight core RNAi machinery genes were mined from WCR, FAW, and SGSB complementary DNA (cDNA) datasets through a series of iterative searches beginning with *Dme* query sequences. Potential candidates were translated in all three coding frames, protein domain structure was predicted and compared with *Dme* sequences, and those exhibiting apparently complete coding DNA sequences (CDSs) and domain structure were selected. Homologues present in each of the three insect pests under investigation are presented in S1 Table, along with translated protein length and accession number. One homologue per pest was found for Drosha, DCR-1, and DCR-2. Multiple versions of potential homologues were identified for Pasha, LOQS, R2D2, AGO1, and AGO2, which may represent products of alternative transcriptional start sites, alternative splicing, or duplicated genes. Regardless of the sources of these differences (i.e. paralogues versus orthologues), for the purposes of this study all versions of the same protein are referred to as isoforms.

### Classification and analysis of putative homologues

**Fig 1:**
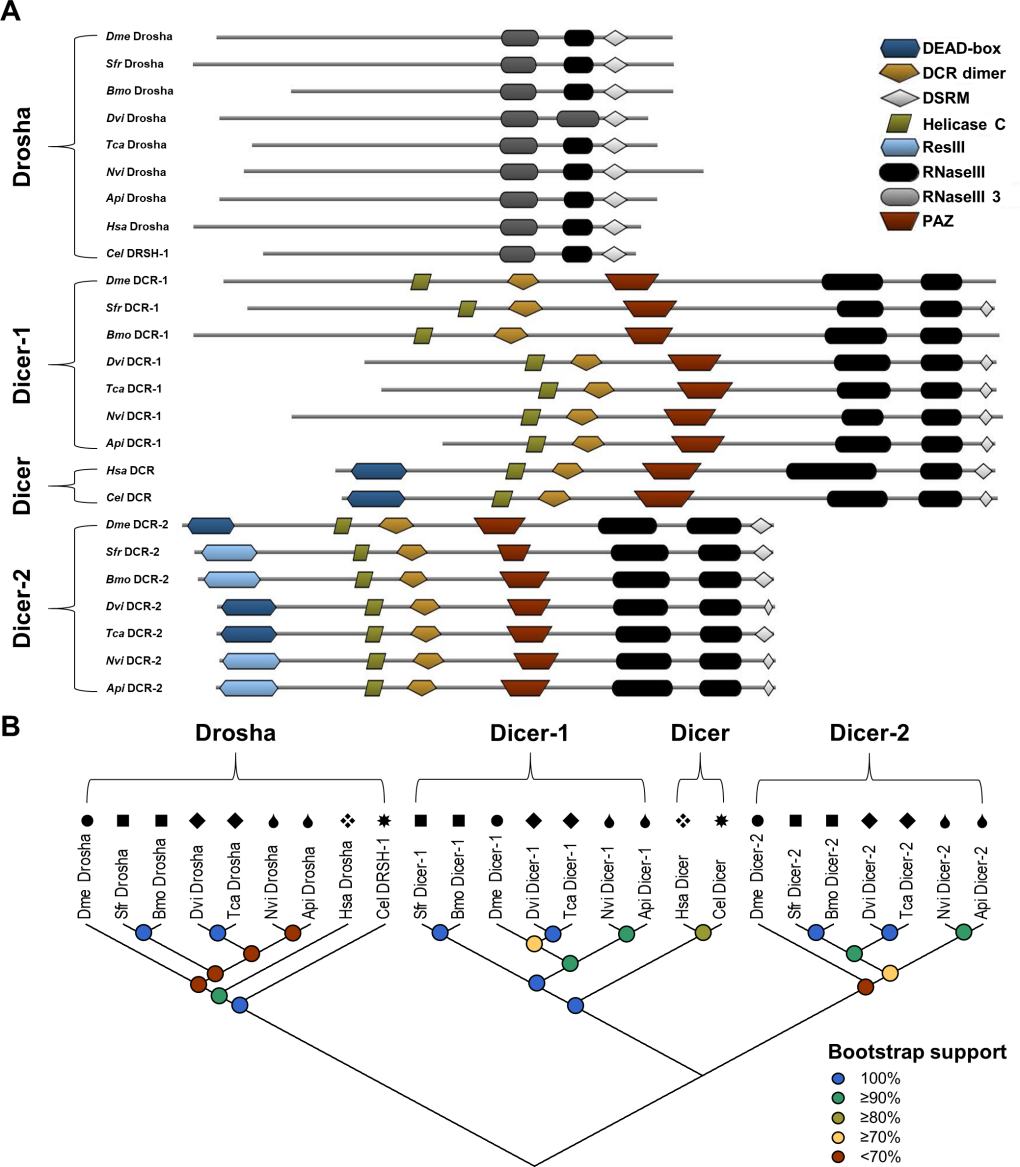
Properties of core mi- and si-RNA pathway RNaseIII-domain containing proteins in WCR, FAW, and SGSB. (A) The predicted protein domains encoded by the Drosha, Dicer, Dicer-1, and Dicer-2 transcripts of *A*. *pisum* (*Api*: XP_003247913.1, XP_001944314.2, XP_016665103.1), *B*. *mori* (*Bmo*: XM_004928209.1, XM_004922309.1, XP_012551309.1), *C*. *elegans* (*Cel*: *NP_492599*.*1*, *NP_498761*.*2*), *D*. *melanogaster* (*Dme*: *NP_477436*.*1*, *NP_524453*.*1*, *NP_523778*.*2*), *D*. *virgifera virgifera* (WCR–*Dvi*: MG225416, MG225417, MG225418), *H*. *sapiens* (*Hsa*: NP_001093882.1, NP_001258211.1), *N*. *viridula* (SGSB–*Nvi*: MG225445, MG225446, MG225447), *S*. *frugiperda* (FAW–*Sfr*: MG225429, MG225430, MG225431), and *T*. *castaneum* (*Tca*: XP_967454.2, XP_008199045.1, XP_008201496.1). Predicted domains include two RNaseIIIs (PF00636 and PF14622), a DCR dimer motif (PF03368), a DSRM (PF00035), a helicase C (PF00271), a PAZ (PF02170), and either a ResIII (PF04851) or DEAD-box helicase (PF00270) domain. E-values for domains predicted in the WCR, FAW, and SGSB proteins range from 4.0×10^−10^ to 1.1×10^−36^, with the exception of the DCR-1 and DCR-2 C-terminal DSRMs. (B) Maximum likelihood phylogenetic tree topology of translated RNaseIII protein-coding sequences (1000 bootstrap replications). Black symbols above each entry indicate phylogenetic order as follows: circles (●) for Diptera, squares (■) for Lepidoptera, diamonds (◆) for Coleoptera, teardrops (💧) for Hemiptera, xrhombus (❖) for Primate, and sunburst (✸) for Rhabditida.

**Fig 2:**
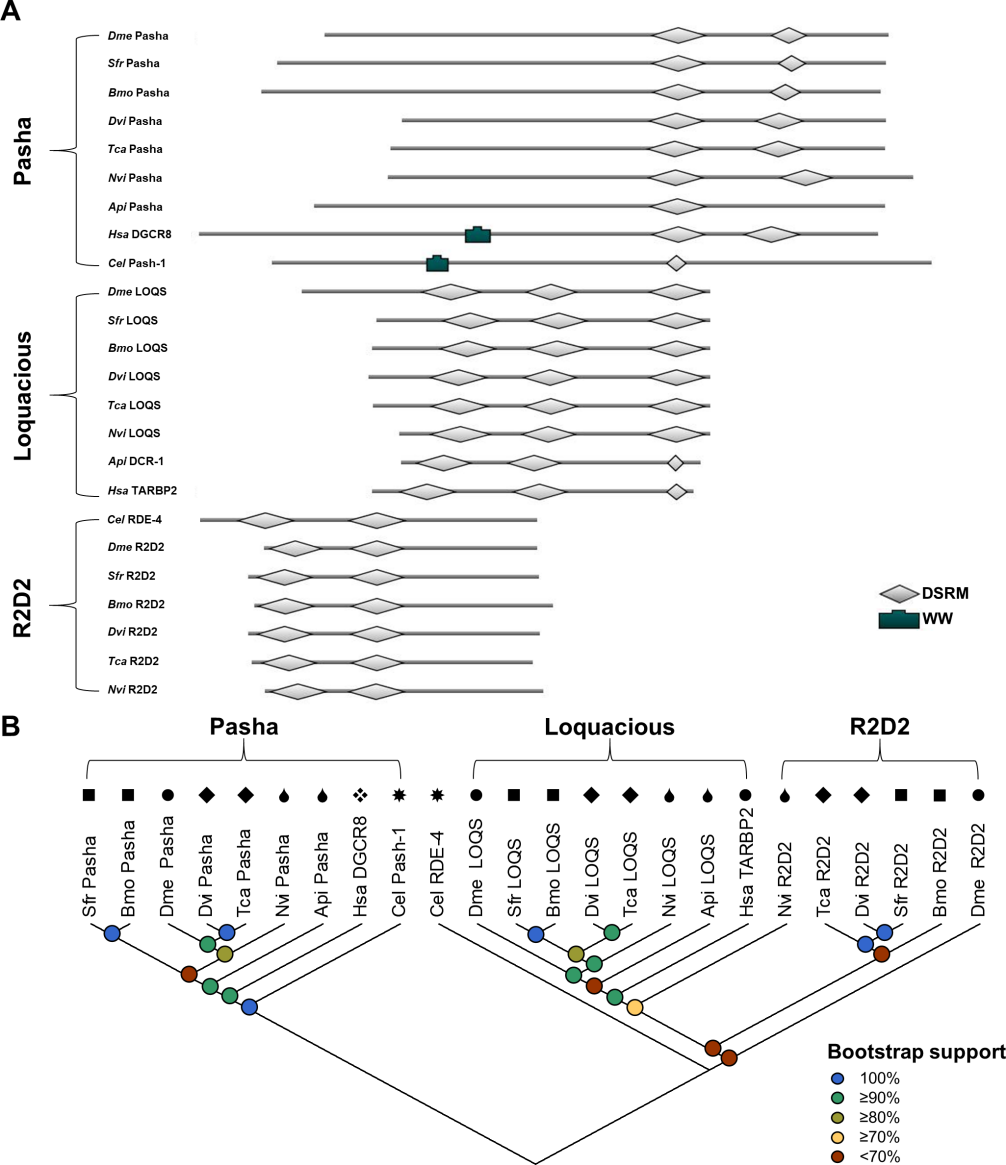
Properties of core mi- and siRNA pathway double-stranded RNA binding proteins in WCR, FAW, and SGSB. (A) The predicted protein domains encoded by the Pasha-PA, LOQS-PB, and R2D2 transcripts of *A*. *pisum* (Api: XP_001947403.1, XP_016657757.1), *B*. *mori* (*Bmo*: XP_012552270.1, XP_012550849.1, NP_001182007.1), *C*. *elegans* (*Cel*: NP_001293461.1, NP_499265.1), *D*. *melanogaster* (*Dme*: NP_651879.1, NP_609646.1, NP_609152.1), *D*. *virgifera virgifera* (WCR–*Dvi*: MG225419, MG225420, MG225423), *H*. *sapiens* (*Has*: NP_073557.3, NP_599150.1), *N*. *viridula* (SGSB–*Nvi*: MG225448, MG225451, MG225453), *S*. *frugiperda* (FAW–*Sfr*: MG225432, MG225435, MG225438), and *T*. *castaneum* (*Tca*: XP_971282.1, XP_966668.1, NP_001128425.1). Predicted domains include two to three DSRMs (PF00035) and a WW (PF00397). E-values for domains predicted in WCR, FAW, and SGSB range from 1.5×10^−3^ to 9.2×10^−15^. (B) Maximum likelihood phylogenetic tree topology of translated dsRBP protein-coding sequences (1000 bootstrap replications). Black symbols above each entry indicate phylogenetic order as follows: circles (●) for Diptera, squares (■) for Lepidoptera, diamonds (◆) for Coleoptera, teardrops (💧) for Hemiptera, xrhombus (❖) for Primate, and sunburst (✸) for Rhabditida.

**Fig 3:**
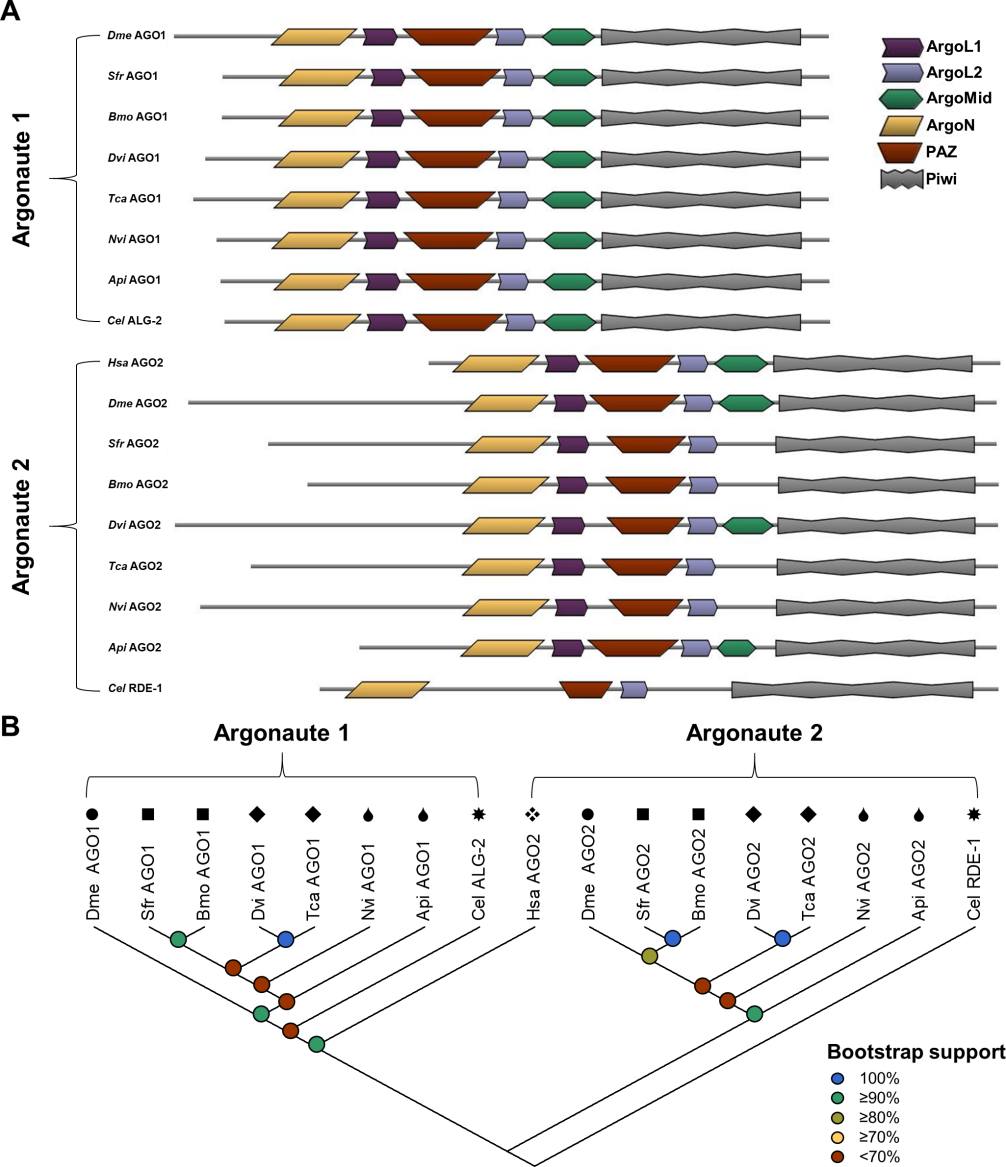
Properties of core mi- and siRNA pathway argonaute proteins in WCR, FAW, and SGSB. (A) The predicted protein domains encoded by the AGO1-PC and AGO2-PB transcripts of *A*. *pisum* (*Api*: XP_003240620.1, XM_001944817.3), *B*. *mori* (*Bmo*: BAF73719.1, XP_012548543.1), *C*. *elegans* (*Cel*: NP_871992.1, NP_741611.1), *D*. *melanogaster* (*Dme*: NP_001246314.1, NP_730054.1), *D*. *virgifera virgifera* (WCR–*Dvi*: MG225424, MG225426), *H*. *sapiens* (*Hsa*: NP_036286.2), *N*. *viridula* (SGSB–*Nvi*: MG225455, MG225456), *S*. *frugiperda* (FAW–*Sfr*: MG225441, MG225443), and *T*. *castaneum* (*Tca*: XP_015837987.1, XP_008192985.1). Predicted domains include an ArgoN (PF16486), an ArgoL1 (PF08699), a PAZ (PF02170), an ArgoL2 (PF16488), an ArgoMid (PF16487), and a Piwi (PF02171). E-values for domains predicted in WCR, FAW, and SGSB range from 1.1×10^−8^ to 1.6×10^−110^. (B) Maximum likelihood phylogenetic tree topology of translated AGO protein-coding sequences (1000 bootstrap replications). Black symbols above each entry indicate phylogenetic order as follows: circles (●) for Diptera, squares (■) for Lepidoptera, diamonds (◆) for Coleoptera, teardrops (💧) for Hemiptera, xrhombus (❖) for Primate, and sunburst (✸) for Rhabditida.

Translated putative isoforms for each protein follow *Dme* nomenclature, which was assigned by conducting a blastp search against a database of corresponding *Dme* proteins. A total of 9 ribonuclease III domain-containing (RNaseIII), 21 dsRBP, and 12 AGO sequences were found across WCR, FAW, and SGSB. A series of bioinformatics tools was then used to examine putative homologues for similarity to each other, to sequences previously reported in related insect species, and to sequences reported from model organisms. *Tribolium castaneum* (*Tca*), *Bombyx mori* (*Bmo*), and *Acyrthosiphon pisum* (*Api*) sequences were included to represent closely-related members of the coleopteran, lepidopteran, and hemipteran insect phylogenetic orders, respectively. *Homo sapiens* (*Has*), *Cel*, and *Dme* sequences serve as well-characterized reference core RNAi proteins, and represent more distant relations to putative homologues from the pests of interest. Final analyses were conducted by partitioning translated reference sequences and candidate homologues into three groups: RNaseIII proteins, dsRBPs, and AGO proteins. To the extent possible, the same putative isoform per protein was selected and used for domain analysis and the reconstruction of phylogenetic trees shown in Figs 1–3. Results of these analyses show that proteins identified in each insect exhibit phylogenetic behavior and domain structure comparable to each other and to reference sequences. Results of domain analyses conducted for all identified sequences are summarized in S2 Table.

### Ribonuclease III domain-containing proteins

Translations of the RNaseIII sequences were scanned against the Pfam database to determine protein domain architecture. Results show that each of the translated pest sequences contain the same number and type of domains as reference sequences (Fig 1A). These sequences display two RNase III domains and—with the exception of *Dme* and *Bmo* DCR-1—a C-terminal dsRNA binding motif (DSRM). The DSRMs of the DCR proteins are predicted with less confidence, showing much higher expect (e-)values (approaching 1) than any of the other domains identified in this study. The DCR proteins also contain a DCR dimer motif, an RNA binding domain common to PIWI, AGO, and Zwille proteins (PAZ), and a helicase C domain. The DCR-2 proteins additionally contain the N-terminal DEAD-box helicase domain consistent with the role of *Dme* DCR-2 translocation along a dsRNA substrate [33]. Under the conditions used in this study for protein domain prediction (see “Methods”), the DEAD-box helicase domain registers as the closely-related bacterial restriction III enzyme domain (ResIII) in the lepidopteran and hemipteran sequences.

Next, parsimony informative sites were identified from an RNaseIII protein multiple sequence alignment (MSA) and used in phylogenetic tree reconstruction. Putative homologues cluster as would be expected for correctly assigned sequences, with Drosha, DCR-1, and DCR-2 from each insect appearing in three distinct protein clades along with all corresponding reference sequences (Fig 1B). The DCR proteins from *Hsa* and *Cel* show a domain structure closer to the siRNA-specific DCR-2, but cluster with the miRNA-specific DCR-1. The WCR, FAW, or SGSB sequences also cluster with high frequency (bootstrap values >91% excluding SGSB Drosha) on the same branch as the reference sequence from their respective phylogenetic order. Overall, length of translated amino acid sequence, domain structure, and phylogenetic analysis agree well with previously reported results for select WCR and FAW RNaseIII sequences [30–32].

#### Double-stranded RNA binding proteins

The dsRBP sequences were analyzed for protein domain arrangement. Each identified pest dsRBP contains two to three DSRMs, in agreement with reference sequence scans (Fig 2A). Pasha, the partner protein of Drosha, is the longest dsRBP and contains two C-terminal DSRMs—with the exception of *Cel* PASH-1. The *Hsa* and *Cel* DGCR8 and PASH-1 sequences are predicted to contain a tryptophan-rich (WW) motif possibly responsible for mediating specific protein-protein interactions with Drosha [12], which does not register in any of the insect sequences. The LOQS-PB sequences each contain three DSRMs, consistent with proposed functions of binding dsRNA (DSRM1 and DSRM2) and interaction with DCR-1 (DSRM3) [7]. Insect-specific R2D2 is the shortest dsRBP and contains two N-terminal DSRMs. A final FAW R2D2 sequence could not be confidently selected from among harvested candidates, though its presence has been reported in FAW ovary-derived Sf21 cells [32]. The sequence analyzed here as FAW R2D2 represents that which agreed most consistently with reference sequences through bioinformatic evaluations utilized during the identification process (described under “Methods”).

All residues of the dsRBP sequences were used to reconstruct phylogenetic relationships. Generally, the newly identified pest sequences cluster within the same clade and on the same branch as the sequence from their closest related reference organism (Fig 2B). The WCR and FAW Pasha and LOQS homologues show bootstrap values >98% for clustering with *Tca* and *Bmo* sequences. Most R2D2 proteins also cluster within a clade separate from LOQS, though there appears to be lower bootstrap support for the distinct separation of these two dsRBP clades than any other protein groups examined within this study. Each SGSB dsRBP proves an exception by appearing on a branch separate from the available *Api* sequences, with R2D2 appearing in a separate clade altogether. Predicted domain arrangement and phylogeny agree with previous reports of the FAW dsRBPs [32].

#### Argonaute proteins

Protein domain analysis of the AGO sequences reveals each WCR, FAW, and SGSB sequence exhibit the same predicted domain structure as reference sequences (Fig 3A). Generally, all sequences include an N-terminal domain of argonaute (ArgoN), two argonaute linker domains (ArgoL1 and ArgoL2), a PAZ domain, a Mid domain of argonaute (ArgoMid), and a Piwi domain. ArgoL1 was not detected in *Cel* RDE-1 and ArgoMid domains were not detected in FAW, SGSB, *Bmo*, and *Tca* AGO2 or *Cel* RDE-1. The failure to detect an ArgoMid domain in some AGO2 sequences could be due to the scan conditions used—such as software package and settings—or deviation from the classically recognized amino acid sequence such that this domain is not being recognized by the scanning algorithms. A true absence would be very unusual, as ArgoMid has been observed in crystal structures to form extensive critical interactions with the Piwi domain, and also to contain residues essential for recognition and binding of the guide RNA 5’ terminal phosphate [34–36].

All residues of the AGO sequences were used in phylogenetic tree reconstruction. The WCR, FAW, and SGSB AGO1 and AGO2 sequences cluster appropriately into each of two clades, and most also appear on the same branch as the relevant reference sequence (Fig 3B). Bootstrap testing gives lower support values for co-clustering of the SGSB and *Api* sequences. Despite being the only human AGO displaying endonucleolytic activity [37], *Hsa* AGO2 clusters with insect AGO1, in agreement with previous observations [38, 39]. The endonucleolytic activity of *Dme* AGO1 is not involved in the canonical role this protein plays regarding silencing of target RNAs [40]. Though domain structure and phylogeny generally agree with previous reports of select WCR and FAW AGO proteins, the WCR AGO2 sequences reported here are longer [30–32]. These length differences are due to a combination of missing sequence and an assembly error that caused up to a 365 residue truncation of the N-terminus in the previously reported sequence [30], likely because of the highly repetitive nature of this region [41, 42]. Additional internal sequence information allowed the error to be identified and manually corrected, resulting in the true full-length WCR AGO2 sequences reported here.

### Expression patterns of core machinery across insect development

**Fig 4:**
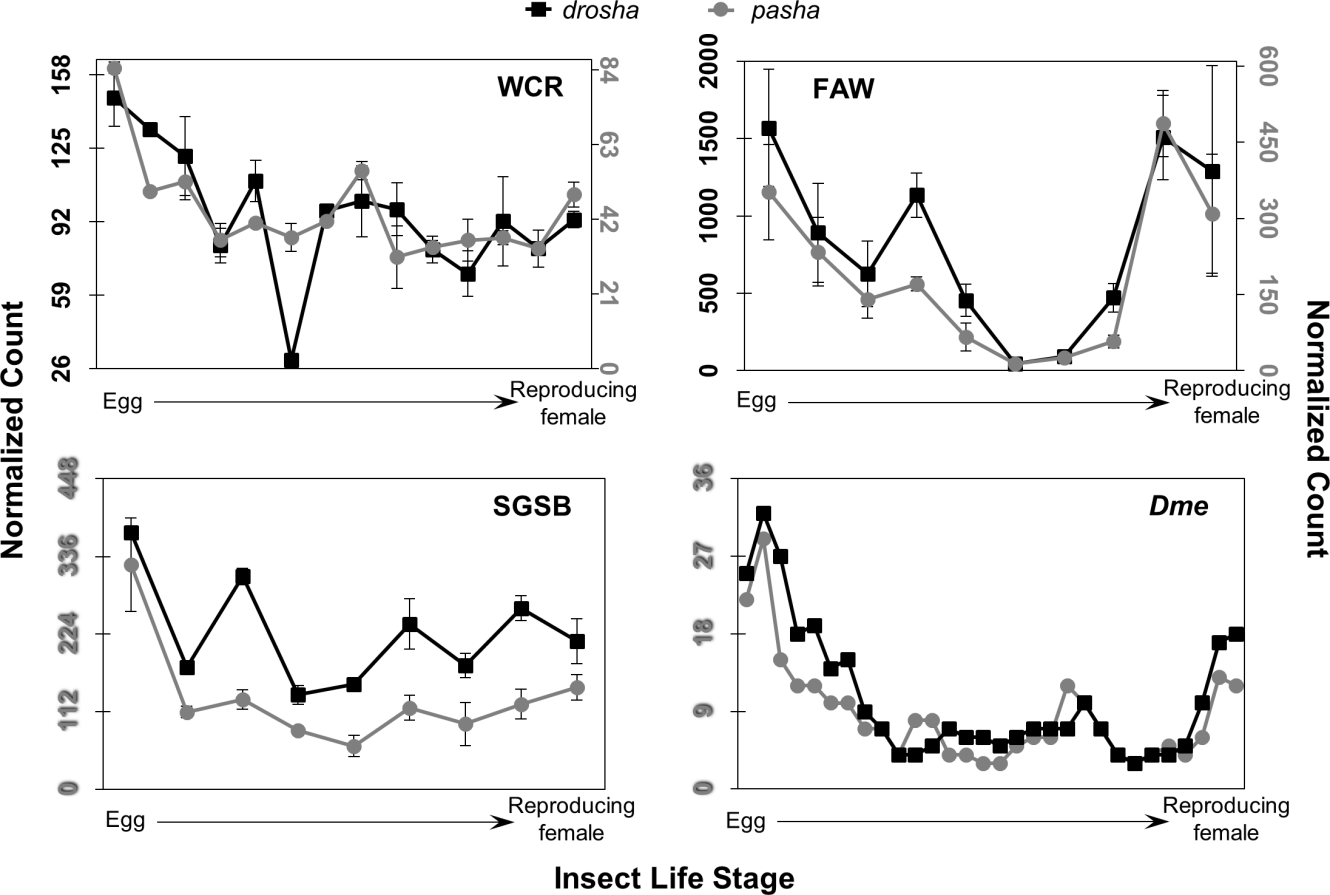
Expression patterns of *drosha* and *pasha* across insect life stage. WCR is shown at top left, FAW at top right, SGSB at bottom left, and *Dme* at bottom right, with *drosha* marked by black squares and *pasha* with grey circles. Normalized count for WCR, FAW, and SGSB transcripts was estimated using RSEM and modeled using DESeq2. The median value across sequencing samples (n = 2 to 4 for pest, n = 30 for *Dme*) is shown, with error bars representing the median absolute deviation (MAD). *Dme* data were obtained from the modENCODE project [33, 34]. Normalized count (in reads per kilobase million) for *Dme* transcripts was generated by adjusting for read depth on a per million scale and length of each target gene in kilobases. Expression of *drosha* is scaled on the left axis in all graphs, and *pasha* on the right for WCR and FAW; axes colors also reflect gene target scaling. *pasha-RA* is shown for WCR and FAW, and–*RAa* for SGSB. Specific *Dme* isoforms are unknown.

**Fig 5:**
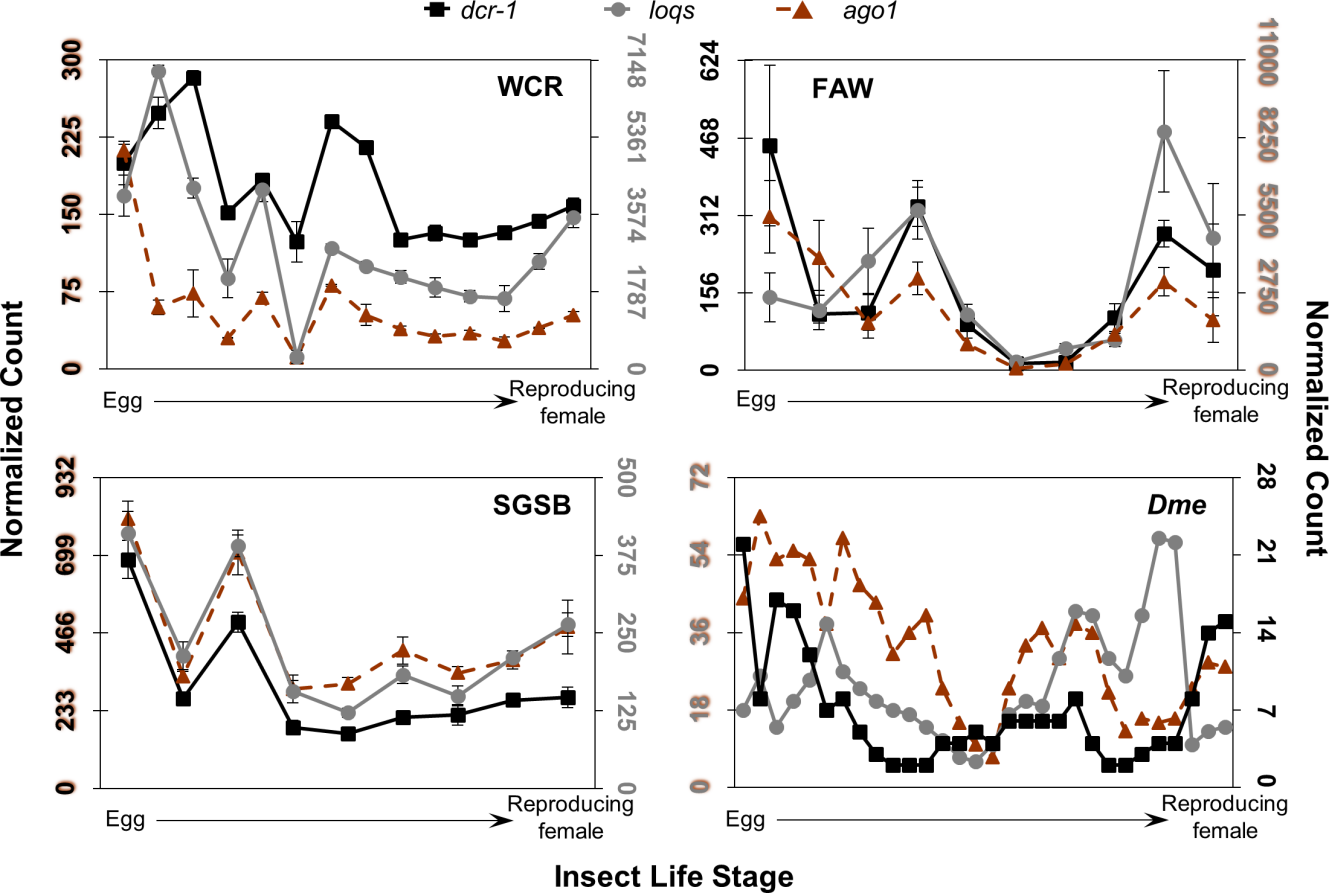
Expression patterns of core miRNA machinery across insect life stage. Normalized count data for each insect are plotted and resulting plots are positioned as in Fig 4. *dcr-1* is shown in black squares, *loqs* in grey circles, and *ago1* in orange triangles with dotted line. The WCR graph scales *loqs-RB* on the left axis, and *dcr-1* and *ago1-RC* on the right. The FAW graph scales *loqs-RBb* and *ago1-RCa* on the left axis, and *dcr-1* on the right; axes colors also reflect gene target scaling. The SGSB graph shows *loqs-RB* and *ago1-RC* isoforms. The *Dme* graph scales *loqs* and *ago1* on the left axis, and *dcr-1* on the right; specific isoforms are unknown.

**Fig 6:**
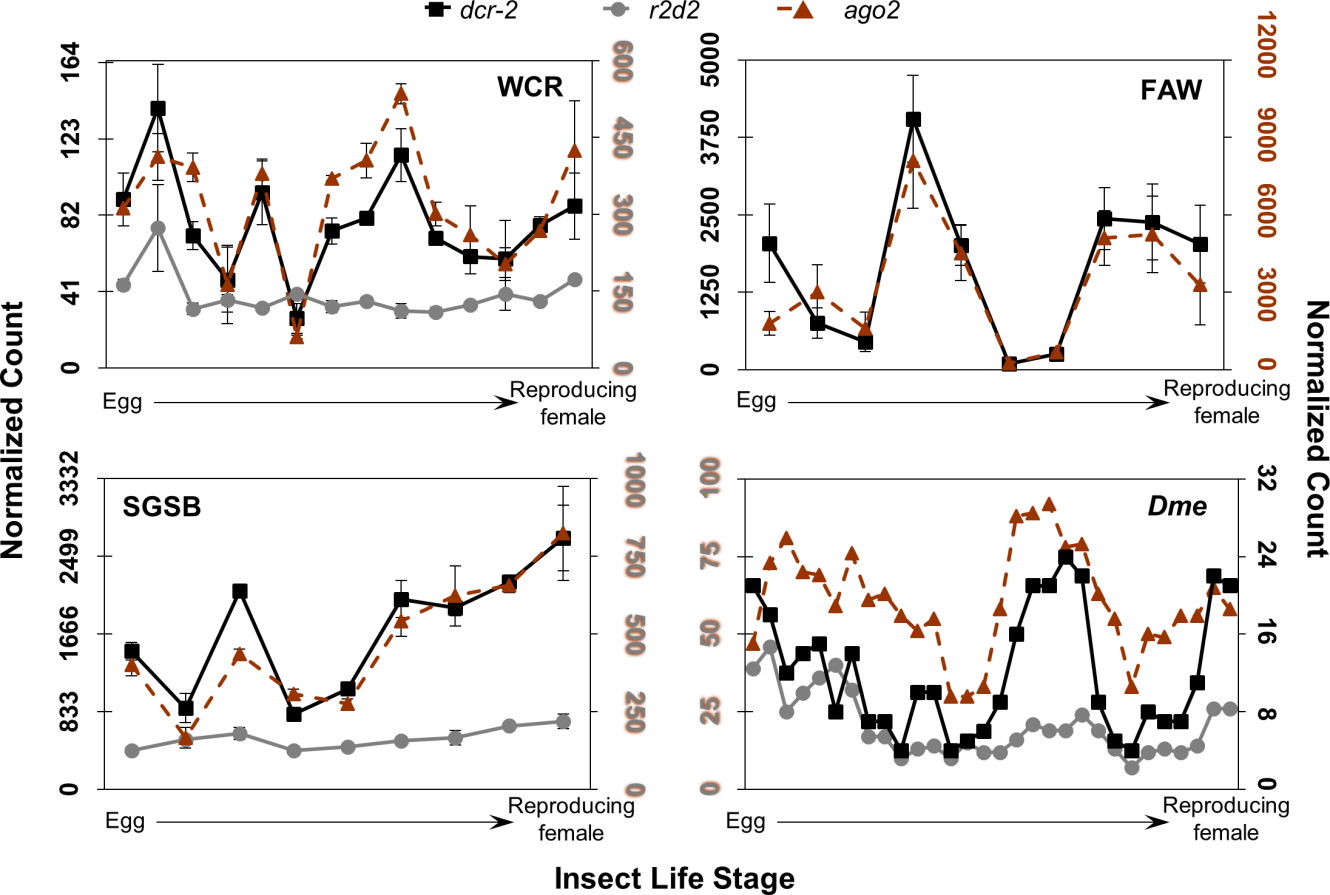
Expression patterns of core siRNA machinery across insect life stages. Normalized count data for each insect are plotted and resulting plots are positioned as in Fig 4. *dcr-2* is shown in black squares, *r2d2* in grey circles, and *ago2* in orange triangles with dotted line. The WCR graph scales *r2d2* and *ago2-RBa* on the left axis, and *dcr-2* on the right. The FAW graph scales *ago2-REa* on the left axis, and *dcr-2* on the right. The SGSB graph scales *dcr-2* on the left axis, and *r2d2-RAa* and *ago2-RB* on the right; axes colors also reflect gene target scaling. The *Dme* graph scales *r2d2* and *ago2* on the left axis, and *dcr-2* on the right; specific isoforms are unknown.

Following identification of core RNAi machinery, expression of these genes was evaluated across the life cycles of WCR, FAW, and SGSB through the use of RNA sequencing (RNA-Seq). As used in this study, the term ‘expression’ refers to normalized transcript abundance levels derived from RNA-Seq experiments. To serve as a point of comparison, expression values of the *Dme* reference machinery were extracted from results of the *Dme* developmental transcriptomes generated as part of the modENCODE project and are also displayed [43, 44]. Details of the 14 (WCR), 10 (FAW), 9 (SGSB), and 30 (*Dme*) life cycle points from which expression data were collected are described in S3 Table. Core machinery of the miRNA pathway for all four insects has been separated into the microprocessor complex of *drosha* and *pasha* (Fig 4), and the downstream genes *dcr-1*, *loqs*, and *ago1* (Fig 5). Core machinery of the siRNA pathway for all four insects are shown together (Fig 6).

Expression patterns of the miRNA machinery show similar trends within insects. The *drosha* and *pasha* transcripts display comparable patterns—these transcripts are most abundant early in the egg but are at lower levels in remaining life stages (Fig 4). Normalized expression values for these transcripts are on average the lowest observed for any of the core machinery, though *drosha* has higher estimated abundance than *pasha*. Expression patterns of the partner proteins *dcr-1* and *loqs* are also generally similar within each insect, though comparison of patterns across insects are more difficult to make (Fig 5). Highest *dcr-1* expression occurs in early to mid-age eggs in all species. While the stage with highest *loqs* levels varies across insects, this transcript reaches the highest value of any core miRNA gene within WCR, FAW and *Dme*. Expression patterns of *ago1* are similar across insects, showing highest levels in early egg followed by lower expression. It also expresses highest of any miRNA transcript in SGSB.

Expression patterns of siRNA machinery transcripts are also consistent within insect (Fig 6). Across insect, these transcripts are variably expressed through life stages rather than peaking early in development. Normalized WCR and *Dme* values are high in egg, dip in late larval and pupal or adult stages, and increase in pre-pupal, early pupal, or actively reproducing adults—especially pregnant females. The FAW transcripts spike in early larval instars and adults. The SGSB transcripts are increasingly expressed from egg to adult, with pregnant females showing the highest expression of *dcr-2* and *ago2*. In WCR, FAW, and *Dme*, *dcr-2* exhibits the lowest expression of the core siRNA transcripts, while in SGSB *r2d2*—the partner protein of *dcr-2* in *Dmel*—is lowest. The most consistently abundant of the siRNA machinery transcripts across the life cycles of WCR, FAW, and *Dme*, is *ago2*, but in SGSB *dcr-2* is generally the most abundant. Expression values for the FAW *r2d2* transcript are not included due to uncertainty in choosing a sequence from among available candidates, though expression of the top candidate sequence was confirmed in whole FAW using reverse transcription polymerase chain reaction (RT-PCR) (S1 Fig). Expression of this transcript was observed in early and late egg, third instar, pupal, and adult female stages—bands were most intense at early egg and third instar; stages that match expression peaks of FAW *dcr-2* and *ago2*.

### Comparison of core machinery expression levels between insect

**Fig 7:**
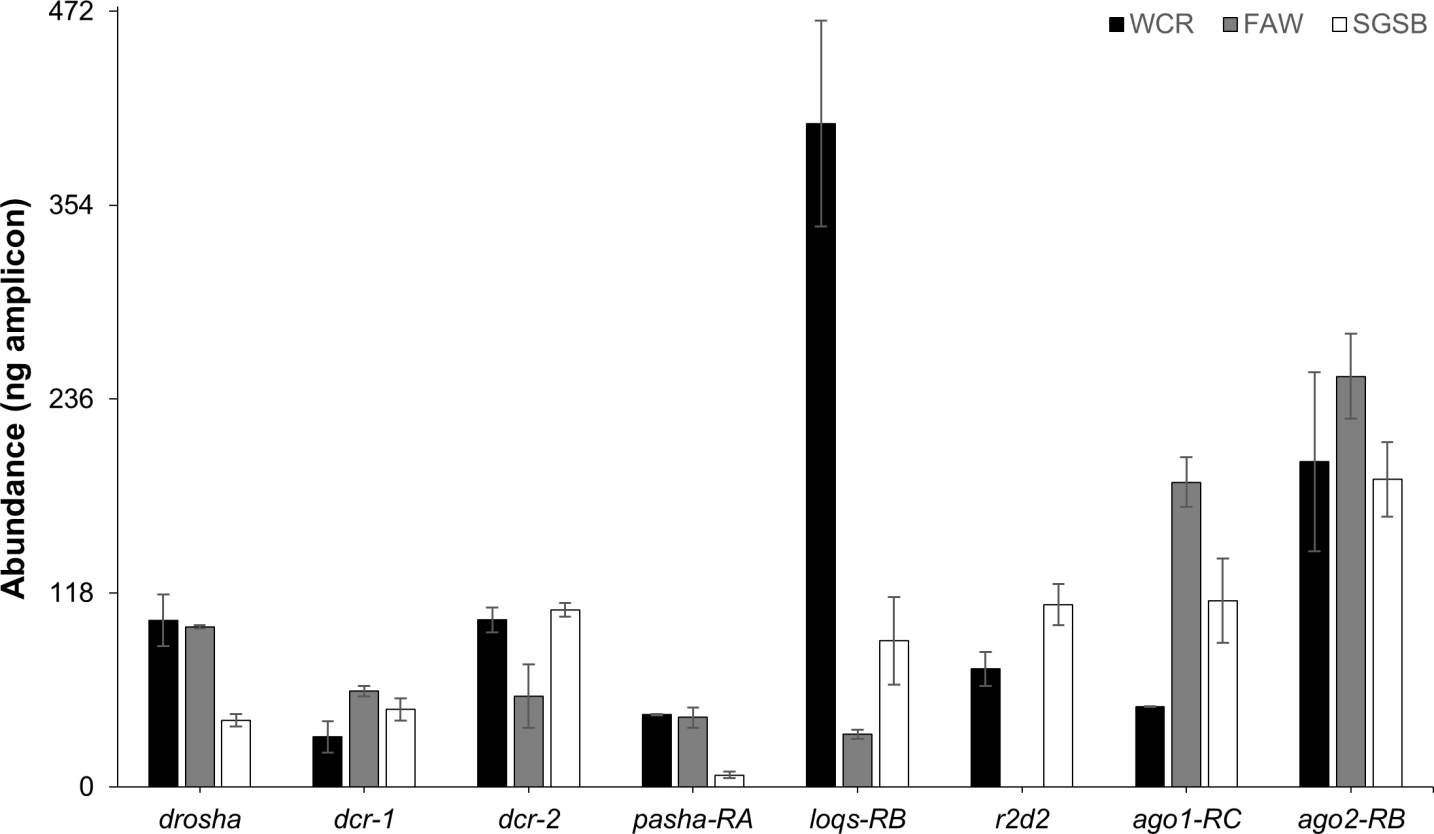
Comparison of core RNAi machinery expression level across WCR, FAW and SGSB. Expression was determined in first instar WCR and FAW and second instar SGSB using optimized semi-quantitative PCR at the end of 31 cycles of amplification, with the exception of SGSB *pasha* and FAW *loqs*. An additional two cycles of amplification were needed to quantify expression of these targets, and values were back-calculated using an assumed 100% PCR reaction efficiency to arrive at the reported values. Median amplicon abundance (n = 3, ± MAD) is shown for the isoform specified, excluding SGSB *r2d2* and WCR *ago2* where primers detected all isoforms. Quantitative expression of the top FAW *r2d2* candidate was not measured.

Expression of core RNAi machinery was directly compared in WCR, FAW, and SGSB to determine baseline levels (Fig 7). Expression of the core RNAi machinery genes in each insect was measured using semi-quantitative RT-PCR with cDNA prepared from samples of the same starting mass and with the same amount of isolated RNA. The life stage chosen for this analysis was midpoint of the first post-hatch stage during which each insect would begin to feed on host crops: first instar for WCR and FAW, second instar for SGSB. Expression of *dcr-1*, *dcr-2*, and *ago2* is similar between insects at this stage, as is *r2d2* in WCR and SGSB (quantitative expression of the top FAW *r2d2* candidate was not measured). A moderately lowered expression of *drosha* and *pasha* in SGSB and *ago1* in WCR is observed when compared with the other two insects. The greatest difference is seen in levels of *loqs* transcript, which are highly elevated in WCR compared with those of FAW or SGSB.

## Discussion

Interest in the use of RNAi-based technology for insect control has increased the necessity of understanding RNAi pathways in non-model insects, especially those of damaging agricultural pests [2]. Examples of such pests include WCR, FAW, and SGSB. These pests also represent phylogenetic orders whose reactions to eRNAi greatly differ. While many coleopterans show robust knockdown of target genes in response to eRNAi, lepidopterans and hemipterans show variable or weak knockdown [26, 27]. Reflecting the response of other beetles, successful control of WCR via knockdown of important genes by transgenic crops expressing dsRNAs was reported ten years ago [6]. In contrast, successful expression knockdown of a FAW gene target via laboratory dsRNA feeding has only been reported twice [45, 46], and has not been reported for SGSB. Differences between characteristics of the core RNAi machinery have not been extensively explored as a potential contributor to the observed responses in these species. Presented here is the first identification and preliminary evaluation of all eight of the core mi- and siRNA pathway genes and their potential isoforms in WCR, FAW, and SGSB. WCR *drosha*, *dcr-1*, *dcr-2*, *ago1*, *ago2*, and FAW *drosha* were also confirmed to agree with previously reported partial sequence [29–32], and the FAW sequences searched for hits against the recently published genome (S1 File) [47]. All sequences were examined and compared with one another and with reference sequences to determine whether differences exist in presence, number of isoforms, protein domain structure, expression pattern, or baseline expression levels. It was hypothesized that variation in these natural characteristics could lead to variation in efficacy of insecticidal RNAs.

At least one sequence homologous to each core component of both the *Dme* mi- and siRNA pathways was identified in WCR, FAW, and SGSB (S1 Table). As defined by *Dme*, these pathways are intact and would be expected to function in generally the same manner if evaluated solely by gene presence. Beyond basic presence of pathway machinery in an insect’s genome, the number of genomic copies has been suggested to confer graded sensitivity to exogenous dsRNAs [48]. The sensitivity of *Tca* appears to be increased beyond the response of several other studied coleopterans, and this insect reportedly has two genomic copies specifically of *ago2* [48]. Homologues of *Dme ago2* have been reported in several lepidopteran and hemipteran pest species [49–51], though a FAW *ago2* homologue was not included in a previous list of RNAi pathway genes detected in Sf21 cells [32]. Interestingly, AGO1 has also been reported to contribute to the response of a coleopteran cell line to exogenous dsRNA [52]. Although exact genomic copy number was not determined in this study, complete absence of any one core RNAi component—including both AGOs—cannot explain the difference in eRNAi response observed between WCR, FAW, and SGSB.

It is well-established in *Dme* that different isoforms exist for core RNAi machinery, and that they impact functioning of the RNAi pathways as discussed below. In an effort to understand differences that may exist between the RNAi pathways in WCR, FAW, and SGSB, it was important to include isoform identification in the current study—though the isoform numbers reported here may not be complete due to limitations of the cDNA databases used. Additionally, isoform designations were assigned based on *in silico* predictions only; they do not guarantee any discrete functionalities—or lack thereof—that have been demonstrated for these proteins in *Dme* and other organisms. Exactly one homologue for each of the RNaseIII proteins was identified in each pest, a result in line with reports from *Dme* and other relevant insects [32, 48–51, 53, 54]. This does not exclude the possibility that different isoforms of these or other core RNAi machinery genes may exist under different conditions, a state which has been observed for mammalian Drosha and DCR [55, 56]. Several different isoforms for the dsRBPs and AGOs were discovered for WCR, FAW, and SGSB, also consistent with the *Dme* RNAi pathway machinery.

Different isoforms of the dsRBP Pasha may be required for localization in either the nucleus or cytoplasm to facilitate distinct functions of Drosha. Most Drosha functions have been found to depend on Pasha [11, 12, 57], and this RNaseIII protein—typically thought to function in the nucleus—has been implicated in the cytoplasmic antiviral response of *Dme* cells [58]. It then follows that Pasha should also likely be present in the cytoplasm under those circumstances. Two versions of Pasha have been reported for *Dme*, which differ from one another at the N-terminus: PA/C and PB (S2 Fig). A nuclear localization signal (NLS) is predicted in the first 50 amino acids for *Dme* Pasha-PA/PC—a region that is absent in the Pasha-PB isoform. This may suggest a nuclear function for one isoform and a cytoplasmic function for the other. Both Pasha isoforms were identified in FAW, with PA containing a predicted N-terminal NLS that is missing in PB (S2 Fig). Only one Pasha isoform was classified in WCR and SGSB. The WCR Pasha was classified by homology as a PB isoform, but is predicted to contain an NLS and was therefore designated a PA isoform (S2 Fig). Three variants of Pasha-PA were found in SGSB (designated PAa, PAb, and PAc); they deviate at the amino acid level in their DSRMs, but non-N-terminal NLSs are predicted in all three (S2 Fig).

Isoforms of the dsRBP LOQS have been demonstrated to play distinct functional roles in *Dme* RNAi pathways. Both the PB and PA isoforms contain three DSRMs, but differ in both their interaction with DCR-1 and expression by sex. The PB isoform exhibits high binding affinity for DCR-1, is the primary dsRBP facilitating dicing of many pre-miRNAs, and shows higher expression than PA in female flies [17]. The PA isoform is lower affinity, can rescue the phenotype of PB-deficient flies but in some cases produces miRNAs of different length and seed sequence, and shows higher expression than PB in male flies [17]. Sequences classified as LOQS-PB and -PA isoforms were found in WCR, FAW, and SGSB. In all three cases, the PA isoform contains a shortened linker region between the second and third DSRMs, consistent with *Dme* LOQS-PA (S2 Table, S3A Fig). In flies this region encodes a motif essential for forming a hydrophobic interface with DCR-1, and its absence in PA leads to lowered affinity and modified miRNA processing [17, 59].

The *Dme* LOQS-PC and -PD isoforms lack the C-terminal third DSRM, and while PC is thought to be an aberrant transcript the protein for which has yet to be detected in any fly stage or tissue, PD is known to interact with DCR-2 [60–62]. One significant difference between FAW and the other two pests is the apparent presence of an isoform that is missing the third DSRM, similar to LOQS-PD (S2 Table, S3B Fig). In the *Dme* PD isoform, the third DSRM is replaced with a region responsible for DCR-2 interaction [60, 61], and the putative FAW LOQS-PD isoform also shows an exchange of the third DSRM for novel sequence. The presence of LOQS-PD has not been confirmed outside of Drosophilidae, and has been proposed to be an adaptation specific to that family [63]. The LOQS-PA isoform appears to fill the role of -PD in *Aedes aegypti* and potentially in other insects by participating in the siRNA pathway with DCR-2, and additionally exerting a regulatory effect on miRNA production [63]. It is unclear whether the putative LOQS-PD transcript in FAW represents a bona fide isoform. In addition to LOQS-PD, the dsRBP R2D2 also interacts with and modifies the activity of DCR-2 in *Dme* [21, 33]. One R2D2 homologue was identified in WCR, but more than one version was detected in FAW and SGSB.

The importance of AGOs to RNAi has been well characterized in model systems (reviewed in [7, 8]), and all three pests in this report appear to have several different isoforms of both AGO1 and AGO2. The isoforms of *Dme* AGO1 and AGO2 differ at the N-terminus, presumably due to alternative transcriptional start sites. This appears to be consistent for putative isoforms identified in WCR, FAW, and SGSB (S4A and S4B Fig). AGO2 isoforms within these species show a high number of N-terminal amino acid differences. The N-terminus of *Dme* AGO2-PB and -PC exhibits a long, unstructured, glutamine-rich repeat region which is absent in the PE isoform. This feature is common across many arthropod AGO2 sequences [41], and indeed it appears in the AGO2 sequences identified for WCR and SGSB (S4B and S4C Fig). Previous reports of WCR AGO2 did not include this repetitive N-terminus, likely due to a combination of missing sequence and assembly errors [29–31]. The FAW AGO2 sequences identified in this study are smaller than those of WCR and SGSB, and assuming no missing sequence, their N-termini do not contain a high proportion of glutamine residues (S4 Table). They instead contain a higher proportion of lysine and glutamic acid residues and were more homologous to the *Dme* AGO2-PE isoform, which completely lacks the glutamine repeat region (S4C Fig, S4 Table). No reliable cDNAs equivalent to AGO2-PE were identified for WCR or SGSB. Although fitting with the known variability of this region even within members of the same genus and species [41, 42], the importance of such differences is not clear. It has been shown that this region interacts with AGO1 early in *Dme* development [64]. Another proposed function is direct interaction with viruses, which could drive its reported rapid evolution [41, 42]. While interesting from the perspective of development and possible adaptation to viral evolution, it is unknown whether these differences—or differences in AGO1 isoforms—would affect insect response to eRNAi.

A source of variation beyond the presence, number of isoforms, and protein domain structure of the core RNAi machinery across WCR, FAW, and SGSB could be their expression levels in each insect. It is possible that differences in expression may promote contrasting responses, even under circumstances where the same proteins exist across species and serve identical functional roles. Examination of expression patterns of the core RNAi machinery across life stages of WCR, FAW, SGSB, and *Dme* reveals surprisingly similar trends (Figs 4–6). Within and across insects, most transcripts whose protein products partner together—and those that cooperate in the same pathway—show similar patterns of expression at the same stage or within a one-stage delay. In several cases, that pattern roughly propagates across species. It is important to note that changes in expression of siRNA factors have been observed upon viral infection and eRNAi in other insects [65–68], and baseline expression across the four insects shown here would not reflect differences in changes occurring in response to various stressors such as ingestion of insecticidal RNA.

In addition to expression patterns across life stage, direct comparison of transcript levels across a field-relevant WCR, FAW, and SGSB life stage revealed one major difference between WCR, an insect with robust response to exogenous dsRNAs, and FAW and SGSB that do not: an increased *loqs* expression (Fig 7). Variations in the roles of LOQS may exist in these insects, as LOQS isoforms are known to perform different functions in both the mi- and siRNA pathways of other insects. Expression data for FAW *r2d2* were not included, but expression of this dsRBP does not differ between WCR and SGSB. Poor expression of *r2d2* has previously been suggested as a potential explanation of the insensitivity of a lepidopteran ovarian cell line to dsRNA [53]. Furthermore, a previous direct comparison of the expression of several core RNAi components in immortalized coleopteran pupal and lepidopteran ovarian cell lines showed universally lower expression in the lepidopteran cells, which was proposed to partially explain the observed discrepancy in dsRNA sensitivity [52]. Results from the current study indicate that expression levels of RNAi genes may not be a consistent source of disparity in whole insects, though *r2d2* does show lower expression relative to the other core machinery genes. It is possible that induction of *r2d2* or specific *loqs* isoforms may occur under conditions of dsRNA challenge; these types of responses have been observed for *dcr-2* and *ago2* in lepidopteran and hemipteran insects, but those studies did not include evaluation of *r2d2* [66, 69, 70]. It is also possible that expression level has fundamentally different effects in each insect that would be undetectable from these data. For example, differences in correlation with translation or intrinsic activities of each insects’ proteins would not be apparent. Expression of RNAi proteins specifically in gut tissues may not be comparable to evaluation using whole organisms; however, this seems improbable considering oral ingestion is a primary route of insect exposure to entomopathogenic viruses for other insects [71]. The expression pattern, transcript, and protein abundance of LOQS isoforms and R2D2 in WCR, FAW, and SGSB must be further evaluated under both baseline conditions as well as under dsRNA challenge.

Recent research suggests nuclease content and dsRNA uptake mechanisms are important factors in determining the effectiveness of eRNAi across different insects [48, 72–76], but limited investigation has occurred on the role the core RNAi machinery may also play. Several differences between the core RNAi machinery of WCR, FAW, and SGSB were identified in the present study. Although relevance of these differences to eRNAi is unknown, based on the information presented here they cannot be ruled out as potential contributors to the differing responses of these insects. Purified proteins for *in vitro* experimentation and whole organisms under conditions of viral or insecticidal dsRNA challenge would assist in parsing the mechanisms and interactions of the core RNAi machinery in these three pests. The information provided here may serve as a basis for such future work.

## Methods

### Homologue identification

Putative homologues for core RNAi machinery from WCR, FAW, and SGSB were retrieved by locally querying internal cDNA databases with *Dme* CDSs and the tblastx algorithm with an e-value cutoff of 1×10^−10^. These internal databases had been previously assembled using Trinity (v. 2.0.6), IDBA-Tran (v. 1.1.1), Velvet-Oases (v. 1.2.10–0.2.08), and/or SOAPdenovo-Trans (v. 1.03) [77–80]. Resulting hits were translated to identify likely CDSs, and those showing adequate length were evaluated by local HMMER3.0 scans in the DoMosaics software package (v. 0.92) with Pfam 31.0 HMM libraries [81, 82]. Translated sequences showing appropriate Pfam domain structure were manually corrected if misassembly was observed (i.e. appearance of two expected domains in two different reading frames with a long region of sequence overlap), and a variety of protein properties such as molecular weight, extinction coefficient, and isoelectric point were analyzed using the pepstats function of EMBOSS Explorer (v. 2.2.0) and Vector NTI Advance (v. 10.3.1) [83, 84]. Final candidates were chosen based on agreement with *Dme* reference sequence protein domain structure and pepstats-estimated properties. If multiple candidate sequences were found containing the same number but a handful of changed non-consecutive amino acids scattered along the length of the peptide, this was attributed to population variation as the available cDNA databases did not always contain inbred lines. In these cases, only one match was selected. Final putative protein isoforms were classified by using the blastp algorithm (v. 2.2.13) with several scoring matrices (PAM30, PAM70, BLOSUM62) specifically against all reported isoforms of the corresponding *Dme* protein. Sequences were matched to a *Dme* isoform based on highest bit score and lowest e-value. For sequences with very close or identical scoring results across more than one *Dme* isoform, discrepancies in peptide length and distinguishing features of MSA were used to differentiate between isoforms. If unique features were unavailable, sequences were classified alphabetically as sub-designations of the parent isoform.

### Final evaluation of core RNAi machinery sequences

Protein domain analysis was conducted using DoMosaics and InterProScan (v. 4.8 with InterPro database 42) with a scan cutoff e-value of 10 [85]. In some cases, multiple domain predictions overlie the same region and domains showing the lowest e-value were selected for display. NLS sequence was predicted using cNLS Mapper with default settings [86]. Multiple sequence alignment was performed using the MUSCLE algorithm in the MEGA7 software package (v. 7.0.21) [87], with default gap penalty settings, maximum iterations set to 10, and clustering method to UPGMB with minimum lambda of 24. Prior to reconstruction of phylogenetic trees, 56 different amino acid substitution models were tested in MEGA7 using a maximum likelihood fit to identify that which gave the lowest Bayesian Information Criterion score for each protein dataset. Trees were then reconstructed in MEGA7 using the maximum likelihood statistical method with Nearest-Neighbor-Interchange (NNI) heuristic and the WAG+G+F, LG+G+I, and LG+G+F substitution models for the RNaseIII, dsRBP, and AGO datasets, respectively [88, 89]. Gaps were included as part of the datasets analyzed, and uncertainty in each tree was estimated using 1000 replications of the bootstrap test.

### RNA sample preparation

Between 10 and ~2500 insects were sourced from colonies maintained within an internal insectary (DuPont Pioneer, Johnston, IA) at the approximate midpoint of each life stage unless otherwise described (S3 Table). Total RNA was isolated from whole flash-frozen WCR of each of 14 life stages by first homogenizing in Buffer RLT with 0.01% PEG using the RNeasy Mini Kit (Qiagen N.V., Hilden, Germany) following manufacturer’s instructions. Directly following column elution, isolated RNAs were DNase-treated using the Ambion TURBO DNA-*free* Kit and associated protocol (Thermo Fisher Scientific, Inc. Waltham, MA). Purified WCR RNAs were checked for quality and quantity using an Agilent 2100 Bioanalyzer (Agilent Technologies, Inc. Santa Clara, CA) with 2100 Expert software (v. B.02.08.SI648). RNAs larger than 200 nts were isolated from whole live FAW and SGSB of each of ten and nine life stages, respectively, using the Ambion miRVana miRNA isolation kit (Thermo Fisher Scientific, Inc.). Directly following column elution, isolated RNAs were DNase-treated for 90 minutes using the RNase-free DNase kit (Qiagen N.V.) and re-purified using the Isolate II RNA Micro Kit (Bioline, London, England), both per manufacturer’s instructions. Purified RNAs were checked for quality and quantity using a Fragment Analyzer (Advanced Analytical Technologies, Inc. Ankeny, IA) with PROSize 2.0 software (v. 1.3.1.1), and then each FAW and SGSB sample was spiked with diluted Ambion ERCC RNA Spike-In Mix 1 (ThermoFisher Scientific, Inc.) at a ratio of 2 μL to 1 μg RNA.

### Next-generation sequencing

Sequencing libraries from purified RNAs were prepared using the TruSeq mRNA-Seq kit with associated protocol (Illumina, Inc., San Diego, CA). Briefly, mRNAs were isolated via attachment to oligo(dT) beads, chemically fragmented to a mean size of 150 nt, and reverse transcribed into cDNA via random hexamer priming. Resulting double-stranded cDNA fragments were end-repaired to create blunt-end fragments, 3’ adenine-tailed, ligated with indexed TruSeq adapters (Illumina, Inc.), and PCR-amplified using TruSeq primers (Illumina, Inc.). Purified PCR-amplified libraries were checked for quality and quantity on a Bioanalyzer DNA 7500 chip (Agilent Technologies, Inc.) with 2100 Expert software before normalization and sample pooling. Sample pools of 10 nM were clustered and sequenced on the HiSeq 2000 (WCR) or 2500 (WCR, FAW, SGSB) system with TruSeq Sequencing By Synthesis Rapid v3 (WCR) or v4 (WCR, FAW, SGSB) chemistry (Illumina, Inc.), as per manufacturer’s instructions. Samples were sequenced single-read, fifty cycles per read, to a minimum depth of five million reads per sample and a target depth of ten million reads per sample.

### RNA-Seq data normalization

Raw sequencing reads were trimmed to remove bases with quality scores less than 13 and sequence tags less than 24 base pairs (bp), after which samples were deconvoluted based on sequenced index identifier. Filtered reads were aligned to transcriptome assembly references using Bowtie 2 (v. 2.2.2), and gene fragment counts were estimated using RSEM (v. 1.3.0) with default settings [90, 91]. Surrogate variable analysis (svaseq) was supervised on selected measurable ERCC sequences as controls to remove batch effects [92, 93], using the following model:

$$g_{ij}=b_{i0}+d_{i}u_{j}+e_{ij}$$

where *g*_*ij*_ is the expression for gene *i* in sample *j*, and *b*_*i0*_, *d*_*i*_*u*_*j*_, and *e*_*ij*_, represent terms for baseline expression, unknown artifact, and measurement error, respectively, as previously described. DESeq2 (v. 1.10.0) was then used with the same ERCC sequences as sample scaling controls (FAW and SGSB) to model gene expression, with independent filtering to optimize power at the 95% confidence level, and with a variance stabilizing transformation to correct for over-dispersion [94], using the following model:

$$K_{ij}∼\mathrm{NB}(\mu_{ij},\alpha_{i})$$

$$\mu_{ij}=s_{j}q_{ij}$$

$$\log_{2}q_{ij}=x_{j.}\beta_{i}$$

Where *K*_*ij*_ is the observed count for gene *i* in sample *j* following a Negative Binomial (NB) distribution, *s*_*j*_ is the sample scale factor according to ERCC controls, *μ*_*ij*_, *α*_*i*_, and *q*_*ij*_ are all parameters fit to the data, *x*_*j*._ is life stage, and *β*_*i*_ contains the log_2_ fold changes for gene *i*. These model-corrected count values were back-transformed and used to generate expression pattern graphs by calculating the median and median absolute deviation for each sequence per sample type. Isoforms shown in Figs 4–6 are those most abundantly expressed in the samples and/or the one for which an available sequence appeared within the reference used for alignment. Reference sequences and raw sequencing reads that aligned to them for all relevant transcripts are available in S2 File. Pre- and post-normalized count data are available in S3 File.

### Semi-quantitative RT-PCR

Pools of RNA from whole insects of each of nine life stages per insect were prepared using the second procedure described above, and concentrations were determined using a NanoDrop 8000 UV-Vis Spectrophotometer (Thermo Fisher Scientific, Inc.) with software (v. 2.3.2). Reverse transcription was carried out using the SuperScript First-Strand Synthesis System for RT-PCR (Thermo Fisher Scientific, Inc.) by loading 125 ng RNA per reaction and following manufacturer-provided instructions for a combination of random hexamer and oligo-dT priming. For detection of FAW *r2d2*, one reaction per life stage was prepared with 1 μL of undiluted cDNA and primers amplifying a 283 bp transcript region. For detection of all other genes, three reactions per life stage per insect were prepared with 1 μL of a 1:10 cDNA dilution and primers amplifying 300 bp of each gene. PCR reactions were conducted using Platinum PCR SuperMix High Fidelity (FAW *r2d2*, Thermo Fisher Scientific, Inc.) or Phusion High-Fidelity PCR Master Mix (all others, Thermo Fisher Scientific, Inc.), according to manufacturer instructions. No template and no reverse-transcriptase controls were also prepared for each primer pair. Thermal cycling proceeded for 40 (FAW *r2d2*) or a target of 31 (all others) cycles in a C1000 Touch instrument (Bio-Rad Laboratories, Inc., Hercules, CA), after which the entirety of each PCR reaction was loaded onto 1.2% agarose gels and electrophoresed at 100 volts for 90 minutes. Size was indicated through use of the ZipRuler Express DNA Ladder 1 (Thermo Fisher Scientific, Inc.), and a four-point standard curve of pure 300 bp DNA was also included on each quantifying gel. Gels were post-stained with SYBR Safe DNA gel stain (Thermo Fisher Scientific, Inc.) and imaged using a FugiFilm Imager LAS-4000 and ImageQuant LAS-4000 software (v. 1.1, General Electric Corp., Boston, MA). Densitometry was performed using Carestream Molecular Imaging software (v. 5.07.23, Bruker Corp., Billerica, MA). Values for core machinery genes were assessed both with and without normalization using several reference genes confirmed by the same semi-quantitative RT-PCR technique to express at a constant level across life stages and insects. Normalization did not change the expression pattern of core machinery genes, and so directly measured values are presented. Primers and thermal cycling conditions are outlined in S5 Table. Densitometric values calculated for the core RNAi machinery are shown in S6 Table.

## Supporting information

S1 Table Putative isoforms of the WCR, FAW, and SGSB core RNAi machinery. Accession numbers for the coding sequences and parameters of translated peptide sequences for each insect are shown. The translated sequences used for additional *in silico* analysis and for which expression data are displayed are marked by asterisks (*); the specific *Dme* isoform associated with expression data is unknown.
(DOCX)

S2 Table Protein domains predicted in isoforms of the WCR, FAW, and SGSB core RNAi machinery. Domains were predicted as described under "Methods". The translated sequences used for additional *in silico* analysis and for which expression data are displayed are marked by asterisks (*).
(DOCX)

S3 Table Description of life cycle stages for insect expression data. The points displayed in expression graphs (Figs 4–6) correspond to each of the stage numbers in this table for each insect. Graphed points begin at stage number 1 on the left and end at the last collected stage on the right. Additional details are included above, such as insect colony used, age within each stage, insect diet, and replicate number included in expression data. Information pertaining to the *Dme* expression data were taken from the following sources: 1) Graveley, B.R., *et al*., The *D*. *melanogaster* transcriptome: modENCODE RNA-Seq data, 2010, Department of Genetics, University of Cambridge: modMine; 2) Graveley, B.R., *et al*., The developmental transcriptome of *Drosophila melanogaster*. Nature, 2011. 471(7339): p. 473–9.
(DOCX)

S4 Table Amino acid composition of insect AGO2 sequences. Sequences analyzed also pictured in S4C Fig up to the start of the *Dme* AGO2-PE isoform. Amino acid composition analysis was conducted with Vector NTI Advance.
(DOCX)

S5 Table Conditions for PCR amplification of insect genes. (DOCX)

S6 Table Expression values for insect genes determined by semi-quantitative RT-PCR. Values are presented as nanograms of amplicon, and were determined from PCR amplification of cDNA prepared using identical masses of insect and isolated RNA.
(DOCX)

S1 Fig Amplification of *r2d2-RAa* in cDNA from nine FAW life stages. A middle segment from the top FAW *r2d2* candidate transcript was amplified using 40 cycles of PCR. The entirety of each reaction was electrophoresed on a 1.2% agarose gel containing SYBR Safe DNA gel stain, along with 5 μL of ZipRuler Express DNA Ladder 1. Strong amplification of the *r2d2* target occurs in the early egg and third instar samples, while fainter bands appear in the late egg, pupal, and adult female samples. This method could not detect target amplification in first instar, sixth instar, adult male, or pregnant female samples.
(TIF)

S2 Fig Features of insect Pasha isoforms. Alignment and nuclear localization signal (NLS) prediction for the Pasha-PA and -PB isoforms for *Dme*, WCR, FAW, and SGSB were performed using ClustalW with default MEGA7 parameters and cNLS Mapper, respectively. Alignments were performed separately by insect to more clearly depict intraspecific sequence differences, which are not easy to visualize in an aggregated alignment. Alignment text colors represent biochemical properties of the different amino acids, and include the following: yellow (A, M, F, I, V, L), olive (C), green (N, Q, S, T, W), aqua (D, E), blue (P), red (R, K), fuchsia (G), teal (H), and lime (Y). Asterisks (*) above the alignment indicate identical residues, and alignment site numbers are shown at the beginning and end of each block. The highest-scoring NLS is indicated above the relevant residues and includes both the type and score. As described within [83], increasing NLS scores represent higher likelihood of nuclear versus cytoplasmic localization: 2≥ exclusively cytoplasmic, 3–5 both nuclear and cytoplasmic, 7–8 preferentially nuclear, 9≤ exclusively nuclear.
(TIF)

S3 Fig Features of insect LOQS isoforms. Alignments were performed using ClustalW with default MEGA7 parameters. Analyses were separated by insect to more clearly depict intraspecific sequence differences, which are not easy to visualize in an aggregated alignment. Alignment text colors represent biochemical properties of the different amino acids, and include the following: yellow (A, M, F, I, V, L), olive (C), green (N, Q, S, T, W), aqua (D, E), blue (P), red (R, K), fuchsia (G), teal (H), and lime (Y). Asterisks (*) above the alignment indicate identical residues, and alignment site numbers are shown at the beginning and end of each block. A) Alignment of *Dme*, WCR, FAW, and SGSB LOQS-PB and -PA isoforms. The amphipathic helix responsible for higher DCR-1 binding affinity exhibited by the *Dme* PB isoform is indicated by arrows above the *Dme* sequences [56]. Red arrows point to residues for which mutations cause detrimental effects to DCR-1 binding [56]. B) Alignment showing the C-termini of the *Dme* and FAW LOQS isoforms.
(TIF)

S4 Fig Features of insect AGO isoforms. Alignments were performed using ClustalW with default MEGA7 parameters. Analyses for A) and B) were separated by insect to more clearly depict intraspecific sequence differences, which are not easy to visualize in an aggregated alignment. Alignment text colors represent biochemical properties of the different amino acids, and include the following: yellow (A, M, F, I, V, L), olive (C), green (N, Q, S, T, W), aqua (D, E), blue (P), red (R, K), fuchsia (G), teal (H), and lime (Y). Asterisks (*) above the alignment indicate identical residues, and alignment site numbers are shown at the beginning and end of each block. A) Differences at the C-termini of *Dme* and WCR AGO1 isoforms. B) Differences at the C-termini of *Dme*, WCR, FAW, and SGSB AGO2 isoforms. C) Alignment of AGO2-PB sequences from *Dme*, WCR, and SGSB, and AGO2-PEa of FAW.
(TIF)

S1 File Search results of FAW sequences against public databases. (XLSX)

S2 File Raw and normalized RNA-Seq count values for insect genes. (TGZ)

S3 File Reference sequences and aligned raw sequencing reads for insect genes. (XLSX)

## References

[1] Anderson JA, Gipmans M, Hurst S, Layton R, Nehra N, Pickett J, et al Emerging Agricultural Biotechnologies for Sustainable Agriculture and Food Security. J Agric Food Chem. 2016;64(2):383–93. Epub 2016/01/21. 10.1021/acs.jafc.5b04543 26785813

[2] Zhang J, Khan SA, Heckel DG, Bock R. Next-Generation Insect-Resistant Plants: RNAi-Mediated Crop Protection. Trends Biotechnol. 2017. Epub 2017/08/22. 10.1016/j.tibtech.2017.04.009 28822479

[3] Napoli C, Lemieux C, Jorgensen R. Introduction of a Chimeric Chalcone Synthase Gene into Petunia Results in Reversible Co-Suppression of Homologous Genes in trans. The Plant cell. 1990;2(4):279–89. Epub 1990/04/01. 10.1105/tpc.2.4.279 12354959 PMC159885

[4] Fire A, Xu S, Montgomery MK, Kostas SA, Driver SE, Mello CC. Potent and specific genetic interference by double-stranded RNA in Caenorhabditis elegans. Nature. 1998;391(6669):806–11. Epub 1998/03/05. 10.1038/35888 9486653

[5] Guo H, Song X, Wang G, Yang K, Wang Y, Niu L, et al Plant-generated artificial small RNAs mediated aphid resistance. PLoS ONE. 2014;9(5):e97410 Epub 2014/05/14. 10.1371/journal.pone.0097410 24819752 PMC4018293

[6] Baum JA, Bogaert T, Clinton W, Heck GR, Feldmann P, Ilagan O, et al Control of coleopteran insect pests through RNA interference. Nat Biotechnol. 2007;25(11):1322–6. Epub 2007/11/06. 10.1038/nbt1359 17982443

[7] Wilson RC, Doudna JA. Molecular mechanisms of RNA interference. Annual review of biophysics. 2013;42:217–39. Epub 2013/05/10. 10.1146/annurev-biophys-083012-130404 23654304 PMC5895182

[8] Ipsaro JJ, Joshua-Tor L. From guide to target: molecular insights into eukaryotic RNA-interference machinery. Nat Struct Mol Biol. 2015;22(1):20–8. Epub 2015/01/08. 10.1038/nsmb.2931 25565029 PMC4450863

[9] Carthew RW, Sontheimer EJ. Origins and Mechanisms of miRNAs and siRNAs. Cell. 2009;136(4):642–55. Epub 2009/02/26. 10.1016/j.cell.2009.01.035 19239886 PMC2675692

[10] Gammon DB, Mello CC. RNA interference-mediated antiviral defense in insects. Curr Opin Insect Sci. 2015;8:111–20. Epub 2015/06/03. 10.1016/j.cois.2015.01.006 26034705 PMC4448697

[11] Denli AM, Tops BB, Plasterk RH, Ketting RF, Hannon GJ. Processing of primary microRNAs by the Microprocessor complex. Nature. 2004;432(7014):231–5. Epub 2004/11/09. 10.1038/nature03049 15531879

[12] Gregory RI, Yan KP, Amuthan G, Chendrimada T, Doratotaj B, Cooch N, et al The Microprocessor complex mediates the genesis of microRNAs. Nature. 2004;432(7014):235–40. Epub 2004/11/09. 10.1038/nature03120 15531877

[13] Lee YS, Nakahara K, Pham JW, Kim K, He Z, Sontheimer EJ, et al Distinct roles for Drosophila Dicer-1 and Dicer-2 in the siRNA/miRNA silencing pathways. Cell. 2004;117:69–81. 15066283 10.1016/s0092-8674(04)00261-2

[14] Bernstein E, Caudy AA, Hammond SM, Hannon GJ. Role for a bidentate ribonuclease in the initiation step of RNA interference. Nature. 2001;409(6818):363–6. Epub 2001/02/24. 10.1038/35053110 11201747

[15] Schwarz DS, Hutvagner G, Du T, Xu Z, Aronin N, Zamore PD. Asymmetry in the assembly of the RNAi enzyme complex. Cell. 2003;115(2):199–208. Epub 2003/10/22. 14567917 10.1016/s0092-8674(03)00759-1

[16] Khvorova A, Reynolds A, Jayasena SD. Functional siRNAs and miRNAs exhibit strand bias. Cell. 2003;115(2):209–16. Epub 2003/10/22. 14567918 10.1016/s0092-8674(03)00801-8

[17] Forstemann K, Tomari Y, Du T, Vagin VV, Denli AM, Bratu DP, et al Normal microRNA maturation and germ-line stem cell maintenance requires Loquacious, a double-stranded RNA-binding domain protein. PLoS biology. 2005;3(7):e236 Epub 2005/05/28. 10.1371/journal.pbio.0030236 15918770 PMC1141267

[18] Saito K, Ishizuka A, Siomi H, Siomi MC. Processing of pre-microRNAs by the Dicer-1-Loquacious complex in drosophila cells. PLoS biology. 2005;3:1202–12. 10.1371/journal.pbio.0030235 PMC1141268 15918769

[19] Miyoshi K, Okada TN, Siomi H, Siomi MC. Characterization of the miRNA-RISC loading complex and miRNA-RISC formed in the Drosophila miRNA pathway. Rna. 2009;15(7):1282–91. Epub 2009/05/20. 10.1261/rna.1541209 19451544 PMC2704077

[20] Matranga C, Tomari Y, Shin C, Bartel DP, Zamore PD. Passenger-strand cleavage facilitates assembly of siRNA into Ago2-containing RNAi enzyme complexes. Cell. 2005;123(4):607–20. Epub 2005/11/08. 10.1016/j.cell.2005.08.044 16271386

[21] Liu Q. R2D2, a Bridge Between the Initiation and Effector Steps of the Drosophila RNAi Pathway. Science. 2003;301:1921–5. 10.1126/science.1088710 14512631

[22] Pham JW, Pellino JL, Lee YS, Carthew RW, Sontheimer EJ. A Dicer-2-dependent 80s complex cleaves targeted mRNAs during RNAi in Drosophila. Cell. 2004;117(1):83–94. Epub 2004/04/07. 15066284 10.1016/s0092-8674(04)00258-2

[23] Fabian MR, Sonenberg N, Filipowicz W. Regulation of mRNA translation and stability by microRNAs. Annual review of biochemistry. 2010;79:351–79. Epub 2010/06/11. 10.1146/annurev-biochem-060308-103103 20533884

[24] Hammond SM, Boettcher S, Caudy AA, Kobayashi R, Hannon GJ. Argonaute2, a link between genetic and biochemical analyses of RNAi. Science. 2001;293(5532):1146–50. Epub 2001/08/11. 10.1126/science.1064023 11498593

[25] Billi AC, Fischer SEJ, Kim JK. Endogenous RNAi pathways in C. elegans. WormBook: The online review of C elegans biology. 2014:1–49. 10.1895/wormbook.1.170.1 PMC4781133 24816713

[26] Kolliopoulou A, Swevers L. Recent progress in RNAi research in Lepidoptera: Intracellular machinery, antiviral immune response and prospects for insect pest control. Current Opinion in Insect Science. 2014;6:28–34. 10.1016/j.cois.2014.09.019 32846666

[27] Christiaens O, Smagghe G. The challenge of RNAi-mediated control of hemipterans. Current Opinion in Insect Science. 2014;6:15–21. 10.1016/j.cois.2014.09.012 32846663

[28] Joga MR, Zotti MJ, Smagghe G, Christiaens O. RNAi Efficiency, Systemic Properties, and Novel Delivery Methods for Pest Insect Control: What We Know So Far. Frontiers in physiology. 2016;7:553 Epub 2016/12/03. 10.3389/fphys.2016.00553 27909411 PMC5112363

[29] Miyata K, Ramaseshadri P, Zhang Y, Segers G, Bolognesi R, Tomoyasu Y. Establishing an in vivo assay system to identify components involved in environmental RNA interference in the western corn rootworm. PLoS ONE. 2014;9:1–15. 10.1371/journal.pone.0101661 PMC4086966 25003334

[30] Velez AM, Khajuria C, Wang H, Narva KE, Siegfried BD. Knockdown of RNA Interference Pathway Genes in Western Corn Rootworms (Diabrotica virgifera virgifera Le Conte) Demonstrates a Possible Mechanism of Resistance to Lethal dsRNA. PLoS ONE. 2016;11(6):e0157520 Epub 2016/06/17. 10.1371/journal.pone.0157520 27310918 PMC4911125

[31] Wu K, Camargo C, Fishilevich E, Narva KE, Chen X, Taylor CE, et al Distinct fitness costs associated with the knockdown of RNAi pathway genes in western corn rootworm adults. PLoS ONE. 2017;12(12):e0190208 Epub 2017/12/22. 10.1371/journal.pone.0190208 29267401 PMC5739497

[32] Ghosh S, Kakumani PK, Kumar A, Malhotra P, Mukherjee SK, Bhatnagar RK. Genome wide screening of RNAi factors of Sf21 cells reveal several novel pathway associated proteins. BMC genomics. 2014;15:775 Epub 2014/09/10. 10.1186/1471-2164-15-775 25199785 PMC4247154

[33] Cenik ES, Fukunaga R, Lu G, Dutcher R, Wang Y, Tanaka Hall TM, et al Phosphate and R2D2 Restrict the Substrate Specificity of Dicer-2, an ATP-Driven Ribonuclease. Molecular Cell. 2011;42:172–84. 10.1016/j.molcel.2011.03.002 21419681 PMC3115569

[34] Yuan YR, Pei Y, Ma JB, Kuryavyi V, Zhadina M, Meister G, et al Crystal structure of A. aeolicus argonaute, a site-specific DNA-guided endoribonuclease, provides insights into RISC-mediated mRNA cleavage. Mol Cell. 2005;19(3):405–19. Epub 2005/08/03. 10.1016/j.molcel.2005.07.011 16061186 PMC4689305

[35] Wang Y, Sheng G, Juranek S, Tuschl T, Patel DJ. Structure of the guide-strand-containing argonaute silencing complex. Nature. 2008;456(7219):209–13. Epub 2008/08/30. 10.1038/nature07315 18754009 PMC4689319

[36] Boland A, Tritschler F, Heimstadt S, Izaurralde E, Weichenrieder O. Crystal structure and ligand binding of the MID domain of a eukaryotic Argonaute protein. EMBO reports. 2010;11(7):522–7. Epub 2010/06/12. 10.1038/embor.2010.81 20539312 PMC2897117

[37] Janowski BA, Huffman KE, Schwartz JC, Ram R, Nordsell R, Shames DS, et al Involvement of AGO1 and AGO2 in mammalian transcriptional silencing. Nat Struct Mol Biol. 2006;13(9):787–92. Epub 2006/08/29. 10.1038/nsmb1140 16936728

[38] Zhang QJ, Luo YJ, Wu HR, Chen YT, Yu JK. Expression of germline markers in three species of amphioxus supports a preformation mechanism of germ cell development in cephalochordates. EvoDevo. 2013;4(1):17 Epub 2013/06/20. 10.1186/2041-9139-4-17 23777831 PMC3735472

[39] Kao D, Lai AG, Stamataki E, Rosic S, Konstantinides N, Jarvis E, et al The genome of the crustacean Parhyale hawaiensis, a model for animal development, regeneration, immunity and lignocellulose digestion. eLife. 2016;5. Epub 2016/11/17. 10.7554/eLife.20062 PMC5111886 27849518

[40] Miyoshi K, Tsukumo H, Nagami T, Siomi H, Siomi MC. Slicer function of Drosophila Argonautes and its involvement in RISC formation. Genes & development. 2005;19(23):2837–48. Epub 2005/11/17. 16287716 10.1101/gad.1370605 PMC1315391

[41] Hain D, Bettencourt BR, Okamura K, Csorba T, Meyer W, Jin Z, et al Natural variation of the amino-terminal glutamine-rich domain in Drosophila argonaute2 is not associated with developmental defects. PLoS ONE. 2010;5(12):e15264 Epub 2011/01/22. 10.1371/journal.pone.0015264 21253006 PMC3002974

[42] Palmer WH, Obbard DJ. Variation and Evolution in the Glutamine-Rich Repeat Region of Drosophila Argonaute-2. G3. 2016;6(8):2563–72. Epub 2016/06/19. 10.1534/g3.116.031880 27317784 PMC4978909

[43] Graveley BR, Brooks AN, Carlson JW, Cherbas L, Choi J, Davis CA, et al The D. melanogaster transcriptome: modENCODE RNA-Seq data Dmel_R6.13 ed. modMine: Department of Genetics, University of Cambridge; 2010.

[44] Graveley BR, Brooks AN, Carlson JW, Duff MO, Landolin JM, Yang L, et al The developmental transcriptome of Drosophila melanogaster. Nature. 2011;471(7339):473–9. Epub 2010/12/24. 10.1038/nature09715 21179090 PMC3075879

[45] Griebler M, Westerlund SA, Hoffmann KH, Meyering-Vos M. RNA interference with the allatoregulating neuropeptide genes from the fall armyworm Spodoptera frugiperda and its effects on the JH titer in the hemolymph. Journal of insect physiology. 2008;54(6):997–1007. Epub 2008/06/11. 10.1016/j.jinsphys.2008.04.019 18541256

[46] Rodriguez-Cabrera L, Trujillo-Bacallao D, Borras-Hidalgo O, Wright DJ, Ayra-Pardo C. RNAi-mediated knockdown of a Spodoptera frugiperda trypsin-like serine-protease gene reduces susceptibility to a Bacillus thuringiensis Cry1Ca1 protoxin. Environmental microbiology. 2010;12(11):2894–903. Epub 2010/06/16. 10.1111/j.1462-2920.2010.02259.x 20545748

[47] Gouin A, Bretaudeau A, Nam K, Gimenez S, Aury JM, Duvic B, et al Two genomes of highly polyphagous lepidopteran pests (Spodoptera frugiperda, Noctuidae) with different host-plant ranges. Sci Rep. 2017;7(1):11816 Epub 2017/09/28. 10.1038/s41598-017-10461-4 28947760 PMC5613006

[48] Tomoyasu Y, Miller SC, Tomita S, Schoppmeier M, Grossmann D, Bucher G. Exploring systemic RNA interference in insects: a genome-wide survey for RNAi genes in Tribolium. Genome biology. 2008;9:R10 10.1186/gb-2008-9-1-r10 18201385 PMC2395250

[49] Gong L, Wang Z, Wang H, Qi J, Hu M, Hu Q. Core RNAi machinery and three Sid-1 related genes in Spodoptera litura (Fabricius). International Journal of Agriculture and Biology. 2015;17:937–44.

[50] Bansal R, Michel AP. Core RNAi machinery and Sid1, a component for systemic RNAi, in the hemipteran insect, Aphis glycines. International journal of molecular sciences. 2013;14:3786–801. 10.3390/ijms14023786 23396108 PMC3588070

[51] Xu HJ, Chen T, Ma XF, Xue J, Pan PL, Zhang XC, et al Genome-wide screening for components of small interfering RNA (siRNA) and micro-RNA (MiRNA) pathways in the brown planthopper, Nilaparvata lugens (Hemiptera: Delphacidae). Insect Molecular Biology. 2013;22:635–47. 10.1111/imb.12051 23937246

[52] Yoon JS, Shukla JN, Gong ZJ, Mogilicherla K, Palli SR. RNA interference in the Colorado potato beetle, Leptinotarsa decemlineata: Identification of key contributors. Insect Biochem Mol Biol. 2016;78:78–88. Epub 2016/10/18. 10.1016/j.ibmb.2016.09.002 27687845

[53] Swevers L, Liu J, Huvenne H, Smagghe G. Search for limiting factors in the RNAi pathway in silkmoth tissues and the Bm5 cell line: the RNA-binding proteins R2D2 and Translin. PLoS ONE. 2011;6(5):e20250 Epub 2011/06/04. 10.1371/journal.pone.0020250 21637842 PMC3102679

[54] Swevers L, Huvenne H, Menschaert G, Kontogiannatos D, Kourti A, Pauchet Y, et al Colorado potato beetle (Coleoptera) gut transcriptome analysis: Expression of RNA interference-related genes. Insect Molecular Biology. 2013;22:668–84. 10.1111/imb.12054 24580832

[55] Dai L, Chen K, Youngren B, Kulina J, Yang A, Guo Z, et al Cytoplasmic Drosha activity generated by alternative splicing. Nucleic acids research. 2016;44(21):10454–66. Epub 2016/07/30. 10.1093/nar/gkw668 27471035 PMC5137420

[56] Flemr M, Malik R, Franke V, Nejepinska J, Sedlacek R, Vlahovicek K, et al A retrotransposon-driven dicer isoform directs endogenous small interfering RNA production in mouse oocytes. Cell. 2013;155(4):807–16. Epub 2013/11/12. 10.1016/j.cell.2013.10.001 24209619

[57] Yeom KH, Lee Y, Han J, Suh MR, Kim VN. Characterization of DGCR8/Pasha, the essential cofactor for Drosha in primary miRNA processing. Nucleic acids research. 2006;34(16):4622–9. Epub 2006/09/12. 10.1093/nar/gkl458 16963499 PMC1636349

[58] Shapiro JS, Schmid S, Aguado LC, Sabin LR, Yasunaga A, Shim JV, et al Drosha as an interferon-independent antiviral factor. Proceedings of the National Academy of Sciences of the United States of America. 2014;111(19):7108–13. Epub 2014/04/30. 10.1073/pnas.1319635111 24778219 PMC4024876

[59] Jakob L, Treiber T, Treiber N, Gust A, Kramm K, Hansen K, et al Structural and functional insights into the fly microRNA biogenesis factor Loquacious. Rna. 2016;22(3):383–96. Epub 2016/01/16. 10.1261/rna.055426.115 26769856 PMC4748816

[60] Hartig JV, Esslinger S, Bottcher R, Saito K, Forstemann K. Endo-siRNAs depend on a new isoform of loquacious and target artificially introduced, high-copy sequences. The EMBO journal. 2009;28(19):2932–44. Epub 2009/08/01. 10.1038/emboj.2009.220 19644447 PMC2760103

[61] Zhou R, Czech B, Brennecke J, Sachidanandam R, Wohlschlegel JA, Perrimon N, et al Processing of Drosophila endo-siRNAs depends on a specific Loquacious isoform. Rna. 2009;15(10):1886–95. Epub 2009/07/29. 10.1261/rna.1611309 19635780 PMC2743050

[62] Fukunaga R, Zamore PD. Chapter Two—Loquacious, a Dicer Partner Protein, Functions in Both the MicroRNA and siRNA Pathways In: Enzymes FGaFTBT-T, editor. Eukaryotic RNases and their Partners in RNA Degradation and Biogenesis, Part B: Academic Press; 2012 p. 37–68.

[63] Haac ME, Anderson MA, Eggleston H, Myles KM, Adelman ZN. The hub protein loquacious connects the microRNA and short interfering RNA pathways in mosquitoes. Nucleic acids research. 2015;43(7):3688–700. Epub 2015/03/15. 10.1093/nar/gkv152 25765650 PMC4402513

[64] Meyer WJ, Schreiber S, Guo Y, Volkmann T, Welte MA, Muller HA. Overlapping functions of argonaute proteins in patterning and morphogenesis of Drosophila embryos. PLoS genetics. 2006;2(8):e134 Epub 2006/08/29. 10.1371/journal.pgen.0020134 16934003 PMC1557783

[65] Campbell CL, Keene KM, Brackney DE, Olson KE, Blair CD, Wilusz J, et al Aedes aegypti uses RNA interference in defense against Sindbis virus infection. BMC microbiology. 2008;8:47 Epub 2008/03/28. 10.1186/1471-2180-8-47 18366655 PMC2278134

[66] Garbutt JS, Reynolds SE. Induction of RNA interference genes by double-stranded RNA; implications for susceptibility to RNA interference. Insect Biochemistry and Molecular Biology. 2012;42:621–8. 10.1016/j.ibmb.2012.05.001 22634162

[67] Niu J, Smagghe G, De Coninck DI, Van Nieuwerburgh F, Deforce D, Meeus I. In vivo study of Dicer-2-mediated immune response of the small interfering RNA pathway upon systemic infections of virulent and avirulent viruses in Bombus terrestris. Insect Biochem Mol Biol. 2016;70:127–37. Epub 2015/12/30. 10.1016/j.ibmb.2015.12.006 26711439

[68] Xie Y-F, Niu J-Z, Jiang X-Z, Yang W-J, Shen G-M, Wei D, et al Influence of various stressors on the expression of core genes of the small interfering RNA pathway in the oriental fruit fly, Bactrocera dorsalis. Insect Science. 2016:1–13. Epub 5/25/2016. 10.1111/1744-7917.12311 28547890

[69] Liu J, Smagghe G, Swevers L. Transcriptional response of BmToll9-1 and RNAi machinery genes to exogenous dsRNA in the midgut of Bombyx mori. Journal of insect physiology. 2013;59(6):646–54. Epub 2013/04/23. 10.1016/j.jinsphys.2013.03.013 23602829

[70] Li H, Guan R, Guo H, Miao X. New insights into an RNAi approach for plant defence against piercing-sucking and stem-borer insect pests. Plant, cell & environment. 2015;38(11):2277–85. Epub 2015/04/02. 10.1111/pce.12546 25828885

[71] Rincón-Castro CD, Ibarra JE. Entomopathogenic viruses In: Rosas-Garcia NM, editor. Biological Control of Insect Pests. USA: Studium Press LLC; 2011 p. 29–64.

[72] Wang K, Peng Y, Pu J, Fu W, Wang J, Han Z. Variation in RNAi efficacy among insect species is attributable to dsRNA degradation in vivo. Insect Biochem Mol Biol. 2016;77:1–9. Epub 2016/07/28. 10.1016/j.ibmb.2016.07.007 27449967

[73] Rocha JJ, Korolchuk VI, Robinson IM, O'Kane CJ. A phagocytic route for uptake of double-stranded RNA in RNAi. PLoS ONE. 2011;6(4):e19087 Epub 2011/05/12. 10.1371/journal.pone.0019087 21559499 PMC3084738

[74] Li X, Dong X, Zou C, Zhang H. Endocytic Pathway Mediates Refractoriness of Insect Bactrocera dorsalis to RNA Interference. Scientific Reports. 2015;5:8700 10.1038/srep08700 25731667 PMC4346973

[75] Cappelle K, de Oliveira CF, Van Eynde B, Christiaens O, Smagghe G. The involvement of clathrin-mediated endocytosis and two Sid-1-like transmembrane proteins in double-stranded RNA uptake in the Colorado potato beetle midgut. Insect Mol Biol. 2016;25(3):315–23. Epub 2016/03/10. 10.1111/imb.12222 26959524

[76] Shukla JN, Kalsi M, Sethi A, Narva KE, Fishilevich E, Singh S, et al Reduced stability and intracellular transport of dsRNA contribute to poor RNAi response in lepidopteran insects. RNA biology. 2016:0. Epub 2016/06/02. 10.1080/15476286.2016.1191728 PMC4962799 27245473

[77] Grabherr MG, Haas BJ, Yassour M, Levin JZ, Thompson DA, Amit I, et al Full-length transcriptome assembly from RNA-Seq data without a reference genome. Nat Biotechnol. 2011;29(7):644–52. Epub 2011/05/17. 10.1038/nbt.1883 21572440 PMC3571712

[78] Peng Y, Leung HC, Yiu SM, Chin FY. IDBA-UD: a de novo assembler for single-cell and metagenomic sequencing data with highly uneven depth. Bioinformatics. 2012;28(11):1420–8. Epub 2012/04/13. 10.1093/bioinformatics/bts174 22495754

[79] Schulz MH, Zerbino DR, Vingron M, Birney E. Oases: robust de novo RNA-seq assembly across the dynamic range of expression levels. Bioinformatics. 2012;28(8):1086–92. Epub 2012/03/01. 10.1093/bioinformatics/bts094 22368243 PMC3324515

[80] Xie Y, Wu G, Tang J, Luo R, Patterson J, Liu S, et al SOAPdenovo-Trans: de novo transcriptome assembly with short RNA-Seq reads. Bioinformatics. 2014;30(12):1660–6. Epub 2014/02/18. 10.1093/bioinformatics/btu077 24532719

[81] Moore AD, Held A, Terrapon N, Weiner J 3rd, Bornberg-Bauer E. DoMosaics: software for domain arrangement visualization and domain-centric analysis of proteins. Bioinformatics. 2014;30(2):282–3. Epub 2013/11/14. 10.1093/bioinformatics/btt640 24222210

[82] Finn RD, Coggill P, Eberhardt RY, Eddy SR, Mistry J, Mitchell AL, et al The Pfam protein families database: towards a more sustainable future. Nucleic acids research. 2016;44(D1):D279–85. Epub 2015/12/18. 10.1093/nar/gkv1344 26673716 PMC4702930

[83] Rice P, Longden I, Bleasby A. EMBOSS: the European Molecular Biology Open Software Suite. Trends in genetics: TIG. 2000;16(6):276–7. Epub 2000/05/29. 10827456 10.1016/s0168-9525(00)02024-2

[84] Lu G, Moriyama EN. Vector NTI, a balanced all-in-one sequence analysis suite. Briefings in bioinformatics. 2004;5(4):378–88. Epub 2004/12/21. 15606974 10.1093/bib/5.4.378

[85] Apweiler R, Attwood TK, Bairoch A, Bateman A, Birney E, Biswas M, et al The InterPro database, an integrated documentation resource for protein families, domains and functional sites. Nucleic acids research. 2001;29(1):37–40. Epub 2000/01/11. 11125043 10.1093/nar/29.1.37 PMC29841

[86] Kosugi S, Hasebe M, Tomita M, Yanagawa H. Systematic identification of cell cycle-dependent yeast nucleocytoplasmic shuttling proteins by prediction of composite motifs. Proceedings of the National Academy of Sciences of the United States of America. 2009;106(25):10171–6. Epub 2009/06/13. 10.1073/pnas.0900604106 19520826 PMC2695404

[87] Kumar S, Stecher G, Tamura K. MEGA7: Molecular Evolutionary Genetics Analysis Version 7.0 for Bigger Datasets. Mol Biol Evol. 2016;33(7):1870–4. Epub 2016/03/24. 10.1093/molbev/msw054 27004904 PMC8210823

[88] Whelan S, Goldman N. A general empirical model of protein evolution derived from multiple protein families using a maximum-likelihood approach. Mol Biol Evol. 2001;18(5):691–9. Epub 2001/04/25. 10.1093/oxfordjournals.molbev.a003851 11319253

[89] Le SQ, Gascuel O. An improved general amino acid replacement matrix. Mol Biol Evol. 2008;25(7):1307–20. Epub 2008/03/28. 10.1093/molbev/msn067 18367465

[90] Langmead B, Salzberg SL. Fast gapped-read alignment with Bowtie 2. Nature methods. 2012;9(4):357–9. Epub 2012/03/06. 10.1038/nmeth.1923 22388286 PMC3322381

[91] Li B, Dewey CN. RSEM: accurate transcript quantification from RNA-Seq data with or without a reference genome. BMC bioinformatics. 2011;12:323 Epub 2011/08/06. 10.1186/1471-2105-12-323 21816040 PMC3163565

[92] Leek J, Johnson W, Parker H, Fertig E, Jaffe A, Storey J, et al. SVA: Surrogate Variable Analysis. R. 2017. Epub 3.26.0.

[93] Leek JT. svaseq: removing batch effects and other unwanted noise from sequencing data. Nucleic acids research. 2014;42(21). Epub 2014/10/09. 10.1093/nar/gku864 PMC4245966 25294822

[94] Love MI, Huber W, Anders S. Moderated estimation of fold change and dispersion for RNA-seq data with DESeq2. Genome Biol. 2014;15(12):550 Epub 2014/12/18. 10.1186/s13059-014-0550-8 25516281 PMC4302049

