# Supplementary material for: Identification and comparison of key RNA interference machinery from western corn rootworm, fall armyworm, and southern green stink bug
Source: PLoS One. 2018 Sep 5;13(9):e0203160. doi: 10.1371/journal.pone.0203160 (PMC6124762; doi:10.1371/journal.pone.0203160)
Supplement: S1 Table — Accession numbers for the coding sequences and parameters of translated peptide sequences for each insect are shown. The translated sequences used for additional in silico analysis and for which expression data are displayed are marked by asterisks (*); the specific Dme isoform associated with expression data is unknown. (DOCX) [file pone.0203160.s001.docx]

| **S1 Table. Putative isoforms of the WCR, FAW, and SGSB core RNAi machinery** | | | | | | | | | | | | | |
| --- | --- | --- | --- | --- | --- | --- | --- | --- | --- | --- | --- | --- | --- |
| **Gene Name** | ***Dme*** | | | | **WCR** | | | **FAW** | | | **SGSB** | | |
|  | **Isoform designation** | | **Peptide length** | **Accession number** | **Isoform designation** | **Peptide length** | **Accession number** | **Isoform designation** | **Peptide length** | **Accession number** | **Isoform designation** | **Peptide length** | **Accession number** |
|  | **Gene** | **Protein** |  |  |  |  |  |  |  |  |  |  |  |
| **Drosha** | RA | PA | 1327 | NM_058088.4 | PA* | 1248 | MG225416 | PA* | 1401 | MG225429 | PA* | 1338 | MG225445 |
| **Dicer-1** | RA | PA | 2249 | NM_079729.3 | PA* | 1840 | MG225417 | PA* | 2175 | MG225430 | PA* | 2070 | MG225446 |
| **Dicer-2** | RA | PA | 1722 | NM_079054.5 | PA* | 1624 | MG225418 | PA* | 1685 | MG225431 | PA* | 1619 | MG225447 |
|  | RB | PB | 1721 |  |  |  |  |  |  |  |  |  |  |
| **Pasha** | RA, RC | PA, PC | 642 | NM_143622.3 | PA* | 551 | MG225419 | PA* | 693 | MG225432 | PAa* | 598 | MG225448 |
|  |  |  |  |  |  |  |  |  |  |  | PAb | 557 | MG225449 |
|  |  |  |  |  |  |  |  |  |  |  | PAc | 562 | MG225450 |
|  | RB | PB | 580 | NM_001276220.1 | - | - | - | PB | 529 | MG225433 | - | - | - |
| **Loquacious** | RB | PB | 465 | NM_135802.4 | PB* | 389 | MG225420 | PBa | 384 | MG225434 | PB* | 354 | MG225451 |
|  |  |  |  |  |  |  |  | PBb* | 380 | MG225435 |  |  |  |
|  | RF | PF | 464 | NM_001298965.1 | - | - | - | - | - | - | - | - | - |
|  | RA | PA | 419 | NM_165041.3 | PA | 338 | MG225421 | PA | 335 | MG225436 | PA | 305 | MG225452 |
|  | RE | PE | 418 | NM_001298964.1 | PE | 324 | MG225422 | - | - | - | - | - | - |
|  | RC | PC | 383 | NM_001038814.3 | - | - | - | - | - | - | - | - | - |
|  | RD | PD | 359 | NM_001201867.2 | - | - | - | PD | 262 | MG225437 | - | - | - |
| **R2D2** | RA, RB, RC | PA, PB, PC | 311 | NM_135308.2 | PA* | 332 | MG225423 | PAa* | 331 | MG225438 | PAa* | 317 | MG225453 |
|  |  |  |  |  |  |  |  | PAb | 324 | MG225439 | PAb | 313 | MG225454 |
|  |  |  |  |  |  |  |  | PAc | 323 | MG225440 |  |  |  |
| **Argonaute 1** | RC, RA, RD | PC, PA, PD | 984 | NM_166021.2 | PC* | 937 | MG225424 | PCa* | 912 | MG225441 | PC* | 921 | MG225455 |
|  |  |  |  |  |  |  |  | PCb | 928 | MG225442 |  |  |  |
|  | RB | PB | 950 | NM_079010.4 | PB | 916 | MG225425 | - | - | - | - | - | - |
| **Argonaute 2** | RB | PB | 1214 | NM_140518.3 | PBa* | 1236 | MG225426 | - | - | - | PB* | 1196 | MG225456 |
|  |  |  |  |  | PBb | 1208 | MG225427 |  |  |  |  |  |  |
|  | RC | PC | 1217 | NM_168626.3 | PC | 1080 | MG225428 | - | - | - | PC | 1044 | MG225457 |
|  | RE | PE | 787 | NM_001274953.1 | - | - | - | PEa* | 1094 | MG225443 | - | - | - |
|  |  |  |  |  |  |  |  | PEb | 969 | MG225444 |  |  |  |
| Accession numbers for the coding nucleotide sequences and parameters of translated peptide sequences for each insect are shown. The translated sequences used for additional *in silico* analysis and for which expression data are displayed are marked by asterisks (*); the specific *Dme* isoform associated with expression data is unknown. | | | | | | | | | | | | | |
