# Supplementary material for: Identification and comparison of key RNA interference machinery from western corn rootworm, fall armyworm, and southern green stink bug
Source: PLoS One. 2018 Sep 5;13(9):e0203160. doi: 10.1371/journal.pone.0203160 (PMC6124762; doi:10.1371/journal.pone.0203160)
Supplement: S2 Table — Domains were predicted as described under "Methods". The translated sequences used for additional in silico analysis and for which expression data are displayed are marked by asterisks (*). (DOCX) [file pone.0203160.s002.docx]

| **S2 Table. Protein domains predicted in isoforms of the WCR, FAW, and SGSB core RNAi machinery** | | | | | | | | | | | | | | | | |
| --- | --- | --- | --- | --- | --- | --- | --- | --- | --- | --- | --- | --- | --- | --- | --- | --- |
| **Species** | **Sequence** | **Domain** | **ArgoL1** | **ArgoL2** | **ArgoMid** | **ArgoN** | **DEAD** | **Dicer_dimer** | **DSRM** | **Helicase_C** | **PAZ** | **Piwi** | **ResIII** | **Ribonuclease III** | |  |
|  |  | **Pfam ID** | **PF08699** | **PF16488** | **PF16487** | **PF16486** | **PF00270** | **PF03368** | **PF00035** | **PF00271** | **PF02170** | **PF02171** | **PF04851** | **PF14622** | **PF00636** |  |
| WCR | Drosha-PA* |  |  |  |  |  |  |  | 1 |  |  |  |  | 2 |  |  |
|  | Dicer-1-PA* |  |  |  |  |  |  | 1 | 1 | 1 | 1 |  |  |  | 2 |  |
|  | Dicer-2-PA* |  |  |  |  |  | 1 | 1 | 1 | 1 | 1 |  |  |  | 2 |  |
|  | Pasha-PA* |  |  |  |  |  |  |  | 2 |  |  |  |  |  |  |  |
|  | Loquacious-PB* | |  |  |  |  |  |  | 3 |  |  |  |  |  |  |  |
|  | Loquacious-PA | |  |  |  |  |  |  | 3 |  |  |  |  |  |  |  |
|  | Loquacious-PE | |  |  |  |  |  |  | 3 |  |  |  |  |  |  |  |
|  | R2D2-PA* |  |  |  |  |  |  |  | 2 |  |  |  |  |  |  |  |
|  | Argonaute 1-PC* | | 1 | 1 | 1 | 1 |  |  |  |  | 1 | 1 |  |  |  |  |
|  | Argonaute 1-PB | | 1 | 1 | 1 | 1 |  |  |  |  | 1 | 1 |  |  |  |  |
|  | Argonaute 2-PBa* | | 1 | 1 | 1 | 1 |  |  |  |  | 1 | 1 |  |  |  |  |
|  | Argonaute 2-PBb | | 1 | 1 | 1 | 1 |  |  |  |  | 1 | 1 |  |  |  |  |
|  | Argonaute 2-PC | | 1 | 1 | 1 | 1 |  |  |  |  | 1 | 1 |  |  |  |  |
| FAW | Drosha-PA* |  |  |  |  |  |  |  | 1 |  |  |  |  | 1 | 1 |  |
|  | Dicer-1-PA* |  |  |  |  |  |  | 1 | 1 | 1 | 1 |  |  |  | 2 |  |
|  | Dicer-2-PA* |  |  |  |  |  |  | 1 | 1 | 1 | 1 |  | 1 |  | 2 |  |
|  | Pasha-PA* |  |  |  |  |  |  |  | 2 |  |  |  |  |  |  |  |
|  | Pasha-PB |  |  |  |  |  |  |  | 2 |  |  |  |  |  |  |  |
|  | Loquacious-PBa | |  |  |  |  |  |  | 3 |  |  |  |  |  |  |  |
|  | Loquacious-PBb* | |  |  |  |  |  |  | 3 |  |  |  |  |  |  |  |
|  | Loquacious-PA | |  |  |  |  |  |  | 3 |  |  |  |  |  |  |  |
|  | Loquacious-PD | |  |  |  |  |  |  | 2 |  |  |  |  |  |  |  |
|  | R2D2-PAa* |  |  |  |  |  |  |  | 2 |  |  |  |  |  |  |  |
|  | R2D2-PAb |  |  |  |  |  |  |  | 2 |  |  |  |  |  |  |  |
|  | R2D2-PAc |  |  |  |  |  |  |  | 2 |  |  |  |  |  |  |  |
|  | Argonaute 1-PCa* | | 1 | 1 | 1 | 1 |  |  |  |  | 1 | 1 |  |  |  |  |
|  | Argonaute 1-PCb | | 1 | 1 | 1 | 1 |  |  |  |  | 1 | 1 |  |  |  |  |
|  | Argonaute 2-PEa* | | 1 | 1 |  | 1 |  |  |  |  | 1 | 1 |  |  |  |  |
|  | Argonaute 2-PEb | | 1 | 1 |  | 1 |  |  |  |  | 1 | 1 |  |  |  |  |
| SGSB | Drosha-PA* |  |  |  |  |  |  |  | 1 |  |  |  |  | 1 | 1 |  |
|  | Dicer-1-PA* |  |  |  |  |  |  | 1 | 1 | 1 | 1 |  |  |  | 2 |  |
|  | Dicer-2-PA* |  |  |  |  |  |  | 1 | 1 | 1 | 1 |  | 1 |  | 2 |  |
|  | Pasha-PAa* |  |  |  |  |  |  |  | 2 |  |  |  |  |  |  |  |
|  | Pasha-PAb |  |  |  |  |  |  |  | 2 |  |  |  |  |  |  |  |
|  | Pasha-PAc |  |  |  |  |  |  |  | 2 |  |  |  |  |  |  |  |
|  | Loquacious-PB* | |  |  |  |  |  |  | 3 |  |  |  |  |  |  |  |
|  | Loquacious-PA | |  |  |  |  |  |  | 3 |  |  |  |  |  |  |  |
|  | R2D2-PAa* |  |  |  |  |  |  |  | 2 |  |  |  |  |  |  |  |
|  | R2D2-PAb |  |  |  |  |  |  |  | 3 |  |  |  |  |  |  |  |
|  | Argonaute 1-PC* | | 1 | 1 | 1 | 1 |  |  |  |  | 1 | 1 |  |  |  |  |
|  | Argonaute 2-PB* | | 1 | 1 |  | 1 |  |  |  |  | 1 | 1 |  |  |  |  |
|  | Argonaute 2-PC | | 1 | 1 | 1 | 1 |  |  |  |  | 1 | 1 |  |  |  |  |
| Domains were predicted as described under "Methods". The translated sequences used for additional *in silico* analysis and for which expression data are displayed are marked by asterisks (*). The reader interested in particular functions that have been described for these domains in other organisms beyond what is discussed within the main text may see the excellent 2013 review by Wilson & Doudna [7]. | | | | | | | | | | | | | | | | |
