# Supplementary material for: Identification and comparison of key RNA interference machinery from western corn rootworm, fall armyworm, and southern green stink bug
Source: PLoS One. 2018 Sep 5;13(9):e0203160. doi: 10.1371/journal.pone.0203160 (PMC6124762; doi:10.1371/journal.pone.0203160)
Supplement: S3 Table — The points displayed in expression graphs (Figs 4–6) correspond to each of the stage numbers in this table for each insect. Graphed points begin at stage number 1 on the left and end at the last collected stage on the right. Additional details are included above, such as insect colony used, age within each stage, insect diet, and replicate number included in expression data. Information pertaining to the Dme expression data were taken from the following sources: 1) Graveley, B.R., et al., The D. melanogaster transcriptome: modENCODE RNA-Seq data, 2010, Department of Genetics, University of Cambridge: modMine; 2) Graveley, B.R., et al., The developmental transcriptome of Drosophila melanogaster. Nature, 2011. 471(7339): p. 473–9. (DOCX) [file pone.0203160.s003.docx]

| **S3 Table. Description of life cycle stages for insect expression data** | | | | | | | | | | | | | | | | | | | | | | | | | | | | | | | | |  |
| --- | --- | --- | --- | --- | --- | --- | --- | --- | --- | --- | --- | --- | --- | --- | --- | --- | --- | --- | --- | --- | --- | --- | --- | --- | --- | --- | --- | --- | --- | --- | --- | --- | --- |
| **Western Corn Rootworm (WCR)** | | | | | | | | | | | | **Southern Green Stinkbug (SGSB)** | | | | | | | | | | | | | | | | | | | | |  |
| ***Diabrotica virgifera virgifera*** | | | | | | | | | | | | ***Nezara viridula*** | | | | | | | | | | | | | | | | | | | | |  |
| **Brookings non-diapausing colony** | | | | | | | | | | | | **DuPont Pioneer internal colony** | | | | | | | | | | | | | | | | | | | | |  |
| *Stage Number* | *Stage Description* | | *Insects per Sample* | | | | *Sample Replicates* | | | | *Stage Number* | | *Stage Description* | | | | | | | | *Insects per Sample* | | | | | | *Sample Replicates* | | | | |  |  |
|  |  |  | *RNA-Seq* | | *RT-qPCR* | | *RNA-Seq* | | *RT-qPCR* | |  | |  | | | | | | | | *RNA-Seq* | | | *RT-qPCR* | | | *RNA-Seq* | | | *RT-qPCR* | |  |  |
| 1 | Early egg | Corn-fed parents, 24 hr | | ~9500 | |  | | 3 | |  | | 1 | | Early Egg | | Soybean material-fed parents, <24 hrs old | | | | | | | ~250 | | |  | | | 4 | |  | | |
| 2 | Mid egg | Corn-fed parents, 6 days | | ~5700 | |  | | 3 | |  | | 2 | | Late Egg | | Soybean material-fed, <24 hrs to hatch | | | | | | | ~250 | | |  | | | 4 | |  | | |
| 3 | Late egg | Corn-fed parents, 12 days | | ~4750 | |  | | 4 | |  | | 3 | | First instar nymph | | Soybean material-fed, midpoint of stage | | | | | | | ~250 | | |  | | | 4 | |  | | |
| 4 | Neonate | First instar larvae, <24 hours | | ~512 | | ~1000 | | 3 | | 3 | | 4 | | Second instar nymph | | Soybean material-fed, midpoint of stage | | | | | | | ~250 | | | ~250 | | | 4 | | 3 | | |
| 5 | First & second instar larva | Mix of corn-fed, midpoint of stages | | ~9 | |  | | 3 | |  | | 5 | | Third instar nymph | | Soybean material-fed, midpoint of stage | | | | | | | 25 | | |  | | | 4 | |  | | |
| 6 | Third instar larva | Corn-fed, midpoint of stage | | ~34 | |  | | 3 | |  | | 6 | | Fifth instar nymph | | Soybean material-fed, midpoint of stage | | | | | | | 25 | | |  | | | 4 | |  | | |
| 7 | Mid pupa | Corn-fed, 5 days post-pupation | | 10 | |  | | 3 | |  | | 7 | | Unmated male | | Male adults, 2 days | | | | | | | 25 | | |  | | | 4 | |  | | |
| 8 | Late pupa | Corn-fed, 10 days post-pupation | | 10 | |  | | 2 | |  | | 8 | | Unmated female | | Female adults, 2 days | | | | | | | 25 | | |  | | | 4 | |  | | |
| 9 | Unmated male | Male adults, 2 days | | 10 | |  | | 3 | |  | | 9 | | Pregnant female | | Female adults, 10 days | | | | | | | 25 | | |  | | | 4 | |  | | |
| 10 | Mated male | Male adults, 5 days | | 10 | |  | | 4 | |  | | **Common Fruit Fly*** | | | | | | | | | | | | | | | | | | | | |  |
| 11 | Late mated male | Male adults, 12 days | | 10 | |  | | 4 | |  | | ***Drosophila melanogaster* (*Dme*)** | | | | | | | | | | | | | | | | | | | | |  |
| 12 | Unmated female | Female adults, 2 days | | 10 | |  | | 3 | |  | | **Strain iso-1** | | | | | | | | | | | | | | | | | | | | |  |
| 13 | Mated female | Female adults, 5 days | | 10 | |  | | 4 | |  | | *Stage Number* | | *Stage Description* | | *Insects per Sample* | | *Sample Replicates* | | *Stage Number* | | | *Stage Description* | | | *Insects per Sample* | | | *Sample Replicates* | | | | |
| 14 | Pregnant female | Female adults, 12 days | | 10 | |  | | 4 | |  | |  |  |  |  |  |  |  |  |  |  |  |  |  |  |  |  |  |  |  |  |  |  |
| **Fall Armyworm (FAW)** | | | | | | | | | |  | | 1 | | Embryo, 0-2 hr | | 1 | | 30 | | 16 | | | Third instar larva, puff stage 1-2 | | | 1 | | | 30 | | | | |
| ***Spodoptera frugiperda*** | | | | | | | | | |  | | 2 | | Embryo, 2-4 hr | | 1 | | 30 | | 17 | | | Third instar larva, puff stage 3-6 | | | 1 | | | 30 | | | | |
| **Mix of vendor colonies: Benzon Research, Chesapeake PERL Biotechnology, and French Agricultural Research** | | | | | | | | | |  | | 3 | | Embryo, 4-6 hr | | 1 | | 30 | | 18 | | | Third instar larva, puff stage 7-9 | | | 1 | | | 30 | | | | |
| *Stage Number* | *Stage Description* | | *Insects per Sample* | | | | *Sample Replicates* | | | | 4 | | Embryo, 6-8 hr | | 1 | | 30 | | 19 | | | White pre-pupa, new | | | 1 | | | 30 | | | | |  |
|  |  |  | *RNA-Seq* | | *RT-qPCR* | | *RNA-Seq* | | *RT-qPCR* | | 5 | | Embryo, 8-10 hr | | 1 | | 30 | | 20 | | | White pre-pupa, 12 hr | | | 1 | | | 30 | | | | |  |
| 1 | Early egg | Diet-fed parents, <24 hrs old | | ~2500 | |  | | 4 | |  | | 6 | | Embryo, 10-12 hr | | 1 | | 30 | | 21 | | | White pre-pupa, 24 hr | | | 1 | | | 30 | | | | |
| 2 | Late egg | Diet-fed parents, <24 hrs to hatch | | ~2500 | |  | | 4 | |  | | 7 | | Embryo, 12-14 hr | | 1 | | 30 | | 22 | | | Pupa, 2 days post-WPP | | | 1 | | | 30 | | | | |
| 3 | First instar larva | Diet-fed, midpoint of stage | | ~1000 | | ~1000 | | 4 | | 3 | | 8 | | Embryo, 14-16 hr | | 1 | | 30 | | 23 | | | Pupa, 3 days post-WPP | | | 1 | | | 30 | | | | |
| 4 | Third instar larva | Diet-fed, midpoint of stage | | 25 | |  | | 4 | |  | | 9 | | Embryo, 16-18 hr | | 1 | | 30 | | 24 | | | Pupa, 4 days post-WPP | | | 1 | | | 30 | | | | |
| 5 | Sixth instar larva | Diet-fed, midpoint of stage | | 25 | |  | | 4 | |  | | 10 | | Embryo, 18-20 hr | | 1 | | 30 | | 25 | | | Adult male, 1 day | | | 1 | | | 30 | | | | |
| 6 | Male pupa | Diet-fed, midpoint of stage | | 25 | |  | | 4 | |  | | 11 | | Embryo, 20-22 hr | | 1 | | 30 | | 26 | | | Adult male, 5 days | | | 1 | | | 30 | | | | |
| 7 | Female pupa | Diet-fed, midpoint of stage | | 25 | |  | | 4 | |  | | 12 | | Embryo, 22-24 hr | | 1 | | 30 | | 27 | | | Adult male, 30 days | | | 1 | | | 30 | | | | |
| 8 | Unmated male | Male adults, ~6 days | | 25 | |  | | 4 | |  | | 13 | | First instar larva | | 1 | | 30 | | 28 | | | Adult female, 1 day | | | 1 | | | 30 | | | | |
| 9 | Unmated female | Female adults, ~6 days | | 25 | |  | | 4 | |  | | 14 | | Second instar larva | | 1 | | 30 | | 29 | | | Adult female, 5 days | | | 1 | | | 30 | | | | |
| 10 | Pregnant female | Female adults, ~6 days | | 25 | |  | | 3 | |  | | 15 | | Third instar larva, 12 hrs old | | 1 | | 30 | | 30 | | | Adult female, 30 days | | | 1 | | | 30 | | | | |
| The points displayed in expression graphs (Figs 4-6) correspond to each of the stage numbers in this table for each insect. Graphed points begin at stage number 1 on the left and end at the last stage collected on the right. One stage per insect was assessed via RT-qPCR (Fig 7). Additional details are included above, such as insect colony used, age within each stage, insect diet, insect per replicate, and replicate number. *****Information pertaining to the *Dme* expression data were taken from the following sources: 1) Graveley, B.R., et al., The *D. melanogaster* transcriptome: modENCODE RNA-Seq data, 2010, Department of Genetics, University of Cambridge: modMine; 2) Graveley, B.R., et al., The developmental transcriptome of *Drosophila melanogaster*. Nature, 2011. 471(7339): p. 473-9. | | | | | | | | | | | | | | | | | | | | | | | | | | | | | | | | |  |
