# Supplementary material for: Identification and comparison of key RNA interference machinery from western corn rootworm, fall armyworm, and southern green stink bug
Source: PLoS One. 2018 Sep 5;13(9):e0203160. doi: 10.1371/journal.pone.0203160 (PMC6124762; doi:10.1371/journal.pone.0203160)
Supplement: S4 Table — Sequences analyzed also pictured in S4C Fig up to the start of the Dme AGO2-PE isoform. Amino acid composition analysis was conducted with Vector NTI Advance. (DOCX) [file pone.0203160.s004.docx]

| **S4 Table. Amino acid composition of insect AGO2 sequences** | | | | | | | | |
| --- | --- | --- | --- | --- | --- | --- | --- | --- |
| **Residue** | ***Dme*** | | **WCR** | | **FAW** | | **SGSB** | |
|  | **Number** | **Mole%** | **Number** | **Mole%** | **Number** | **Mole%** | **Number** | **Mole%** |
| A = Ala | 9 | 2.11 | 32 | 7.18 | 35 | 11.4 | 21 | 5.13 |
| C = Cys | 0 | 0.00 | 0 | 0.00 | 2 | 0.65 | 0 | 0.00 |
| D = Asp | 6 | 1.41 | 7 | 1.57 | 7 | 2.28 | 6 | 1.47 |
| E = Glu | 12 | 2.81 | 13 | 2.92 | 35 | 11.4 | 11 | 2.69 |
| F = Phe | 1 | 0.23 | 0 | 0.00 | 2 | 0.65 | 4 | 0.98 |
| G = Gly | 96 | 22.5 | 40 | 8.97 | 15 | 4.89 | 30 | 7.34 |
| H = His | 12 | 2.81 | 7 | 1.57 | 2 | 0.65 | 5 | 1.22 |
| I = Ile | 3 | 0.70 | 4 | 0.90 | 6 | 1.95 | 7 | 1.71 |
| K = Lys | 14 | 3.28 | 26 | 5.83 | 35 | 11.4 | 15 | 3.67 |
| L = Leu | 8 | 1.87 | 11 | 2.47 | 13 | 4.24 | 9 | 2.20 |
| M = Met | 1 | 0.23 | 4 | 0.90 | 9 | 2.93 | 4 | 0.98 |
| N = Asn | 3 | 0.70 | 6 | 1.35 | 5 | 1.63 | 8 | 1.96 |
| P = Pro | 34 | 7.96 | 74 | 16.6 | 48 | 15.6 | 65 | 15.9 |
| Q = Gln | 161 | 37.7 | 135 | 30.3 | 16 | 5.21 | 154 | 37.7 |
| R = Arg | 27 | 6.32 | 19 | 4.26 | 11 | 3.58 | 28 | 6.85 |
| S = Ser | 18 | 4.22 | 31 | 6.95 | 28 | 9.12 | 16 | 3.91 |
| T = Thr | 5 | 1.17 | 22 | 4.93 | 20 | 6.52 | 10 | 2.45 |
| V = Val | 2 | 0.47 | 10 | 2.24 | 14 | 4.56 | 10 | 2.45 |
| W = Trp | 2 | 0.47 | 5 | 1.12 | 2 | 0.65 | 5 | 1.22 |
| Y = Tyr | 13 | 3.04 | 0 | 0.00 | 2 | 0.65 | 1 | 0.24 |
| Z = Glx | 0 | 0.00 | 0 | 0.00 | 0 | 0.00 | 0 | 0.00 |
| Sequences analyzed also pictured in S4C Fig up to the start of the *Dme* AGO2-PE isoform. Amino acid composition analysis was conducted with Vector NTI Advance. | | | | | | | | |
