# Supplementary material for: Identification and comparison of key RNA interference machinery from western corn rootworm, fall armyworm, and southern green stink bug
Source: PLoS One. 2018 Sep 5;13(9):e0203160. doi: 10.1371/journal.pone.0203160 (PMC6124762; doi:10.1371/journal.pone.0203160)
Supplement: S5 Table — (DOCX) [file pone.0203160.s005.docx]

| **S5 Table. Conditions for PCR amplification of insect genes** | | | | | |
| --- | --- | --- | --- | --- | --- |
| **Species** | **Sequence Name** | **Sequence** | **Species** | **Sequence Name** | **Sequence** |
| WCR | *drosha* Forward | GACTCCCACCATCATACATTT | WCR | *loqs-RB* Forward | CATCACTCCCAAATACGAACT |
| WCR | *drosha* Reverse | GGAAGTTCTCGAACGAATCT | WCR | *loqs-RB* Reverse | GACATGCACATCTCTTGGAG |
| FAW | *drosha* Forward | GAGAGTGAAGATGAATGTTTGG | FAW | *loqs-RB* Forward | CACCTTCTTACCATGCTGAG |
| FAW | *drosha* Reverse | CAAGAAGTTTGTTGGTGGTG | FAW | *loqs-RB* Reverse | GATGTTTGTGAAACTGCGAC |
| SGSB | *drosha* Forward | GAATCACTATTTCTCCTCCCA | SGSB | *loqs-RB* Forward | ACGAAACCGAAAATGAAGAG |
| SGSB | *drosha* Reverse | GGGCACAACCATTAGAATCA | SGSB | *loqs-RB* Reverse | GGAATTGTGACACTTTGATG |
| WCR | *dcr-1* Forward | TATTCAATCCGGAAGCTTCA | WCR | *r2d2* Forward | TCTGTTAACTTCGTAGCTCCTT |
| WCR | *dcr-1* Reverse | ATGTAGCGTACTCTGAGCCA | WCR | *r2d2* Reverse | TGCACACATTCGATCTCTGA |
| FAW | *dcr-1* Forward | TTGAGCTGAAGATACTCACGG | FAW | *r2d2* Forward | AAAACAGTGTGAATTAGATGCTGG |
| FAW | *dcr-1* Reverse | TTTTCGAGTTGAATAGTGGG | FAW | *r2d2* Reverse | GAGGACACTCTAATAATAAATCCTG |
| SGSB | *dcr-1* Forward | CTGATGAACAAGACGATTGC | SGSB | *r2d2* Forward | TGTGAAAGTGGAACTCATGAAC |
| SGSB | *dcr-1* Reverse | TTCTTTGTCTCCAACCCCTA | SGSB | *r2d2* Reverse | AACAAACACATGGGCAGTTA |
| WCR | *dcr-2* Forward | GGTTGTAAAAGAAGAAGACGTG | WCR | *ago1-RC* Forward | CTCTCGCCAGTCTTGTTACC |
| WCR | *dcr-2* Reverse | GTGAGGTAAATCGGGAAATC | WCR | *ago1-RC* Reverse | GAAATGGTTCGCTTTGAGAC |
| FAW | *dcr-2* Forward | CCAGCTATAATGGATACACCC | FAW | *ago1-RC* Forward | CCATGAAGGAAGACCCATAA |
| FAW | *dcr-2* Reverse | TATAACGCTCGTCGAAATCA | FAW | *ago1-RC* Reverse | TTGATGCTGACACGAAACAC |
| SGSB | *dcr-2* Forward | TGACAGTTGAATCTGCTATTCC | SGSB | *ago1-RC* Forward | CAGAGTCTGCAATGTTACAAGA |
| SGSB | *dcr-2* Reverse | CGTCTTGAACTTCATTTCCA | SGSB | *ago1-RC* Reverse | ATCTCTCTTTCCCGATCAGG |
| WCR | *pasha-RA* Forward | TGAAGAAGCAACCCACGTAT | WCR | *ago2* Forward | CGGTTTTTCAGTTTCTAGCG |
| WCR | *pasha-RA* Reverse | CGGTAGTTTTGGCACAAAAT | WCR | *ago2* Reverse | GATGTCCATATTTCTGTCACCA |
| FAW | *pasha-RA* Forward | GAACCACTTCGAGGTGCTAC | FAW | *ago2-RB* Forward | GATGAGTCTACTTGTAAAGAGGAAC |
| FAW | *pasha-RA* Reverse | TGGCTGTTCTCGCAGTAGTC | FAW | *ago2-RB* Reverse | TTTAAGGGGATCTGCTGTCT |
| SGSB | *pasha* Forward | CATCGTTTCCTGATGGAACCA | SGSB | *ago2-RB* Forward | AACAAAGGCAAGAACATGCT |
| SGSB | *pasha* Reverse | TTTCCAATGAAGCTTTCGCA | SGSB | *ago2-RB* Reverse | GTCGAGGAGGTCCCTGTTGT |
| **PCR Conditions - FAW R2D2** | | | **PCR Conditions - all others** | | |
| *Step* | *Temp (^o^C)* | *Time (min:sec)* | *Step* | *Temp (^o^C)* | *Time (min:sec)* |
| Platinum PCR SuperMix High Fidelity | | | Phusion High Fidelity PCR Master Mix | | |
| Incubation | 94 | 2:00 | Incubation | 98 | 0:30 |
| Denature | 94 | 0:30 | Denature | 98 | 0:05 |
| Anneal | 47 | 0:30 | Anneal | 58.5 | 0:30 |
| Extend | 68 | 1:00 | Extend | 72 | 0:10 |
| Repeat cycle from 2 x39 (total 40 cycles) | | | Repeat cycle from 2 x30 (total 31 cycles) | | |
| Final Elongation | 68 | 10:00 | Final Elongation | 72 | 5:00 |
| Cool & Hold | 4 | hold | Cool & Hold | 4 | hold |
