# Supplementary material for: Identification and comparison of key RNA interference machinery from western corn rootworm, fall armyworm, and southern green stink bug
Source: PLoS One. 2018 Sep 5;13(9):e0203160. doi: 10.1371/journal.pone.0203160 (PMC6124762; doi:10.1371/journal.pone.0203160)
Supplement: S6 Table — Values are presented as nanograms of amplicon, and were determined from PCR amplification of cDNA prepared using identical masses of insect and isolated RNA. (DOCX) [file pone.0203160.s006.docx]

| **S6 Table. Expression values for insect genes determined by semi-quantitative RT-PCR** | | | | | | | | | | | | |
| --- | --- | --- | --- | --- | --- | --- | --- | --- | --- | --- | --- | --- |
| **Gene** | **WCR** | | | | **FAW** | | | | **SGSB** | | | |
|  | **Life stage** | **Rep1** | **Rep2** | **Rep3** | **Life stage** | **Rep1** | **Rep2** | **Rep3** | **Life stage** | **Rep1** | **Rep2** | **Rep3** |
| *drosha* | First instar | 52.4 | 101 | 117 | First instar | 97.5 | 98.5 | 90.4 | Second instar | 74.1 | 36.8 | 40.5 |
| *dcr-1* |  | 20.6 | 30.3 | 43.3 |  | 61.3 | 52.1 | 58.1 |  | 47.0 | 22.0 | 53.9 |
| *dcr-2* |  | 93.8 | 101 | 150 |  | 35.8 | 55.2 | 77.9 |  | 98.3 | 108 | 112 |
| *pasha* |  | 43.3 | 43.9 | 45.7 |  | 28.7 | 48.4 | 42.2 |  | 5.15 | 9.11 | 9.8 |
| *loqs* |  | 442 | 505 | 603 |  | 29.0 | 35.0 | 31.9 |  | 88.7 | 115 | 55.7 |
| *r2d2* |  | 85.9 | 71.7 | 61.3 |  | Not determined | | |  | 127 | 111 | 98.2 |
| *ago1* |  | 30.5 | 48.9 | 48.7 |  | 170 | 273 | 185 |  | 151 | 113 | 87.7 |
| *ago2* |  | 198 | 123 | 252 |  | 224 | 295 | 250 |  | 93.1 | 210 | 187 |
| Values are presented as ng amplicon, and were determined from PCR amplification of cDNA prepared using identical masses of insect and isolated RNA. | | | | | | | | | | | | |
