# Supplementary figures and images for: Identification and comparison of key RNA interference machinery from western corn rootworm, fall armyworm, and southern green stink bug
Source: PLoS One. 2018 Sep 5;13(9):e0203160. doi: 10.1371/journal.pone.0203160 (PMC6124762; doi:10.1371/journal.pone.0203160)

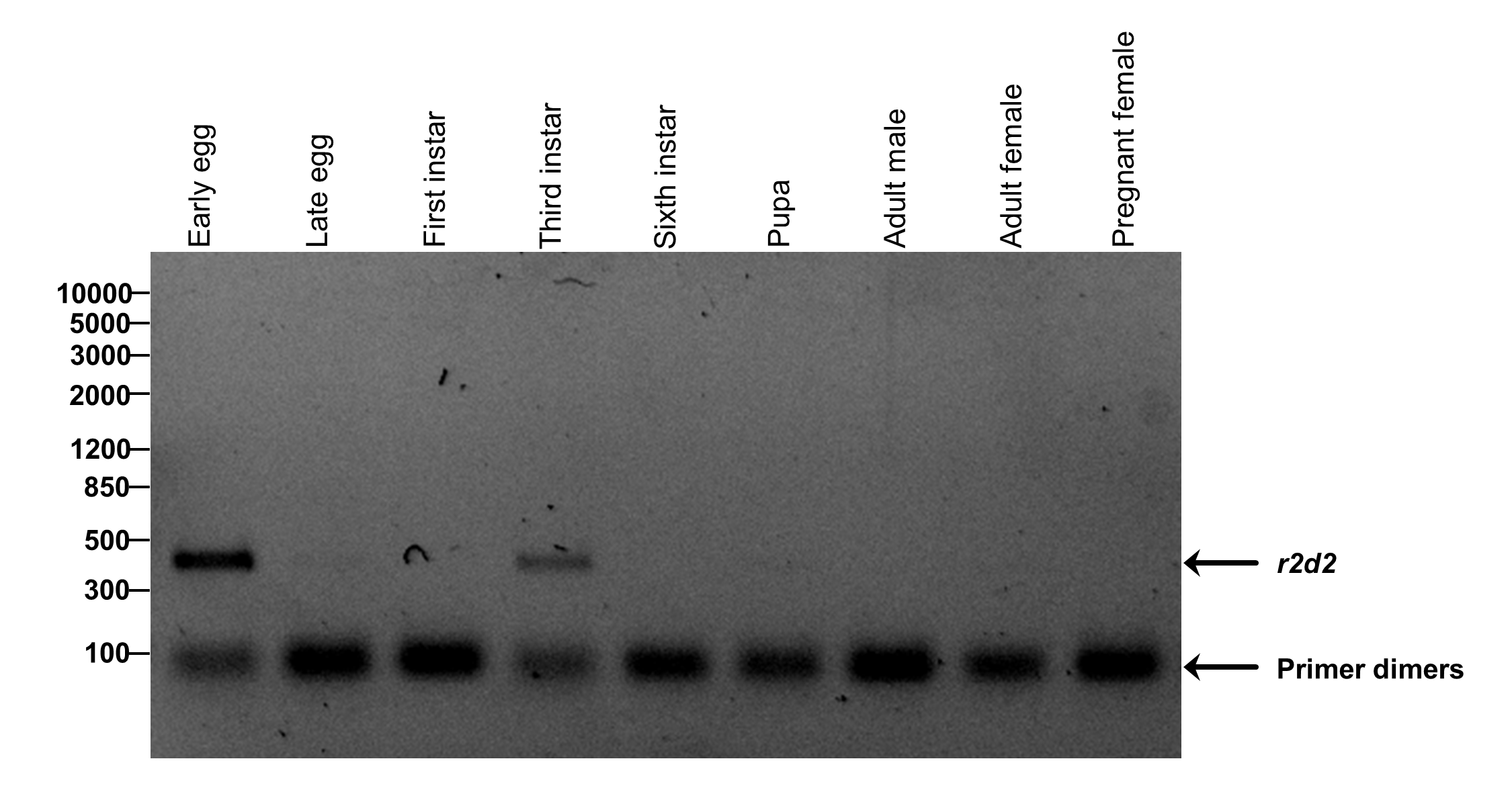

Supplement: S1 Fig — A middle segment from the top FAW r2d2 candidate transcript was amplified using 40 cycles of PCR. The entirety of each reaction was electrophoresed on a 1.2% agarose gel containing SYBR Safe DNA gel stain, along with 5 μL of ZipRuler Express DNA Ladder 1. Strong amplification of the r2d2 target occurs in the early egg and third instar samples, while fainter bands appear in the late egg, pupal, and adult female samples. This method could not detect target amplification in first instar, sixth instar, adult male, or pregnant female samples. (TIF) [file pone.0203160.s007.tif]

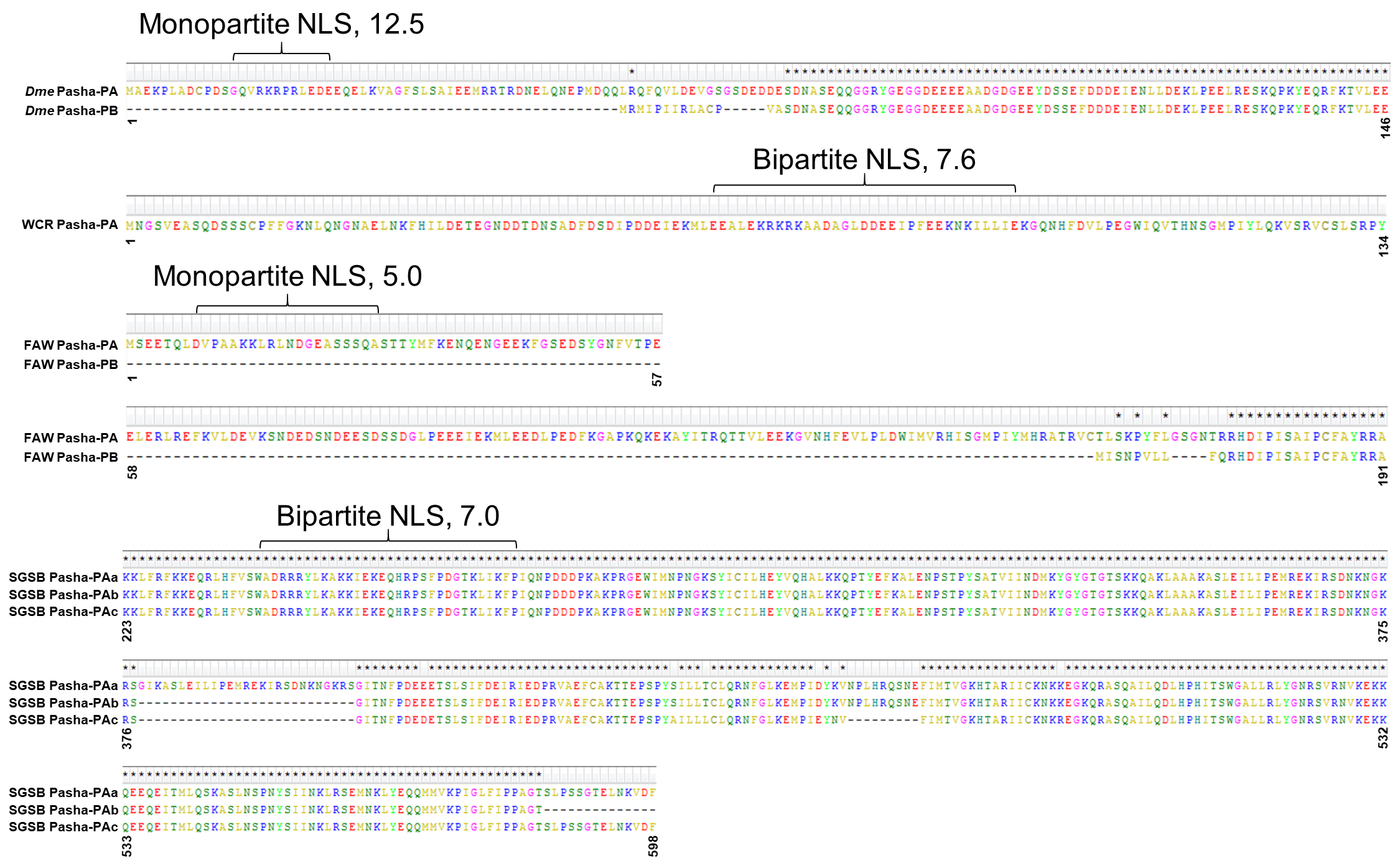

Supplement: S2 Fig — Alignment and nuclear localization signal (NLS) prediction for the Pasha-PA and -PB isoforms for Dme, WCR, FAW, and SGSB were performed using ClustalW with default MEGA7 parameters and cNLS Mapper, respectively. Alignments were performed separately by insect to more clearly depict intraspecific sequence differences, which are not easy to visualize in an aggregated alignment. Alignment text colors represent biochemical properties of the different amino acids, and include the following: yellow (A, M, F, I, V, L), olive (C), green (N, Q, S, T, W), aqua (D, E), blue (P), red (R, K), fuchsia (G), teal (H), and lime (Y). Asterisks (*) above the alignment indicate identical residues, and alignment site numbers are shown at the beginning and end of each block. The highest-scoring NLS is indicated above the relevant residues and includes both the type and score. As described within [83], increasing NLS scores represent higher likelihood of nuclear versus cytoplasmic localization: 2≥ exclusively cytoplasmic, 3–5 both nuclear and cytoplasmic, 7–8 preferentially nuclear, 9≤ exclusively nuclear. (TIF) [file pone.0203160.s008.tif]

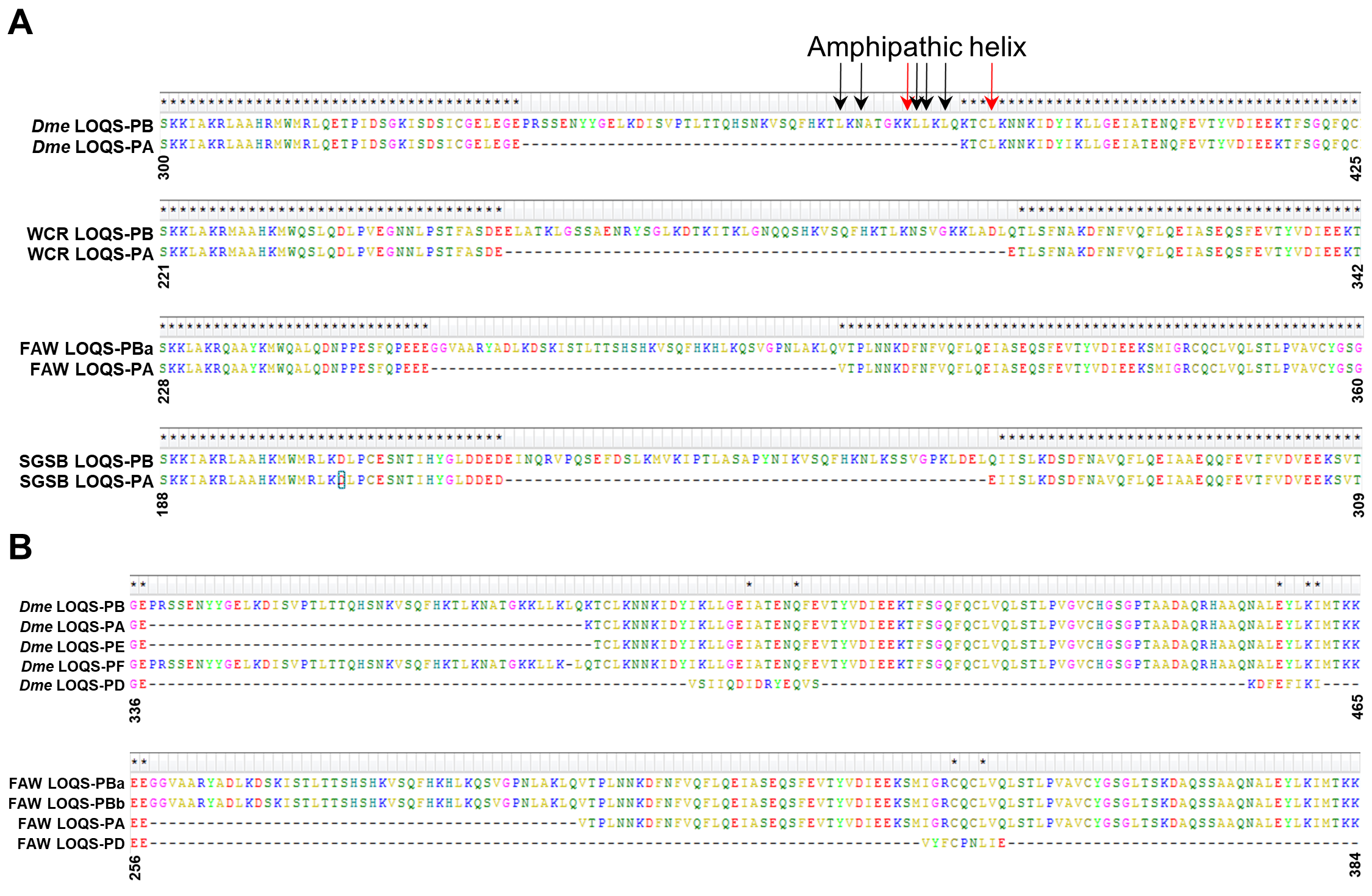

Supplement: S3 Fig — Alignments were performed using ClustalW with default MEGA7 parameters. Analyses were separated by insect to more clearly depict intraspecific sequence differences, which are not easy to visualize in an aggregated alignment. Alignment text colors represent biochemical properties of the different amino acids, and include the following: yellow (A, M, F, I, V, L), olive (C), green (N, Q, S, T, W), aqua (D, E), blue (P), red (R, K), fuchsia (G), teal (H), and lime (Y). Asterisks (*) above the alignment indicate identical residues, and alignment site numbers are shown at the beginning and end of each block. A) Alignment of Dme, WCR, FAW, and SGSB LOQS-PB and -PA isoforms. The amphipathic helix responsible for higher DCR-1 binding affinity exhibited by the Dme PB isoform is indicated by arrows above the Dme sequences [56]. Red arrows point to residues for which mutations cause detrimental effects to DCR-1 binding [56]. B) Alignment showing the C-termini of the Dme and FAW LOQS isoforms. (TIF) [file pone.0203160.s009.tif]

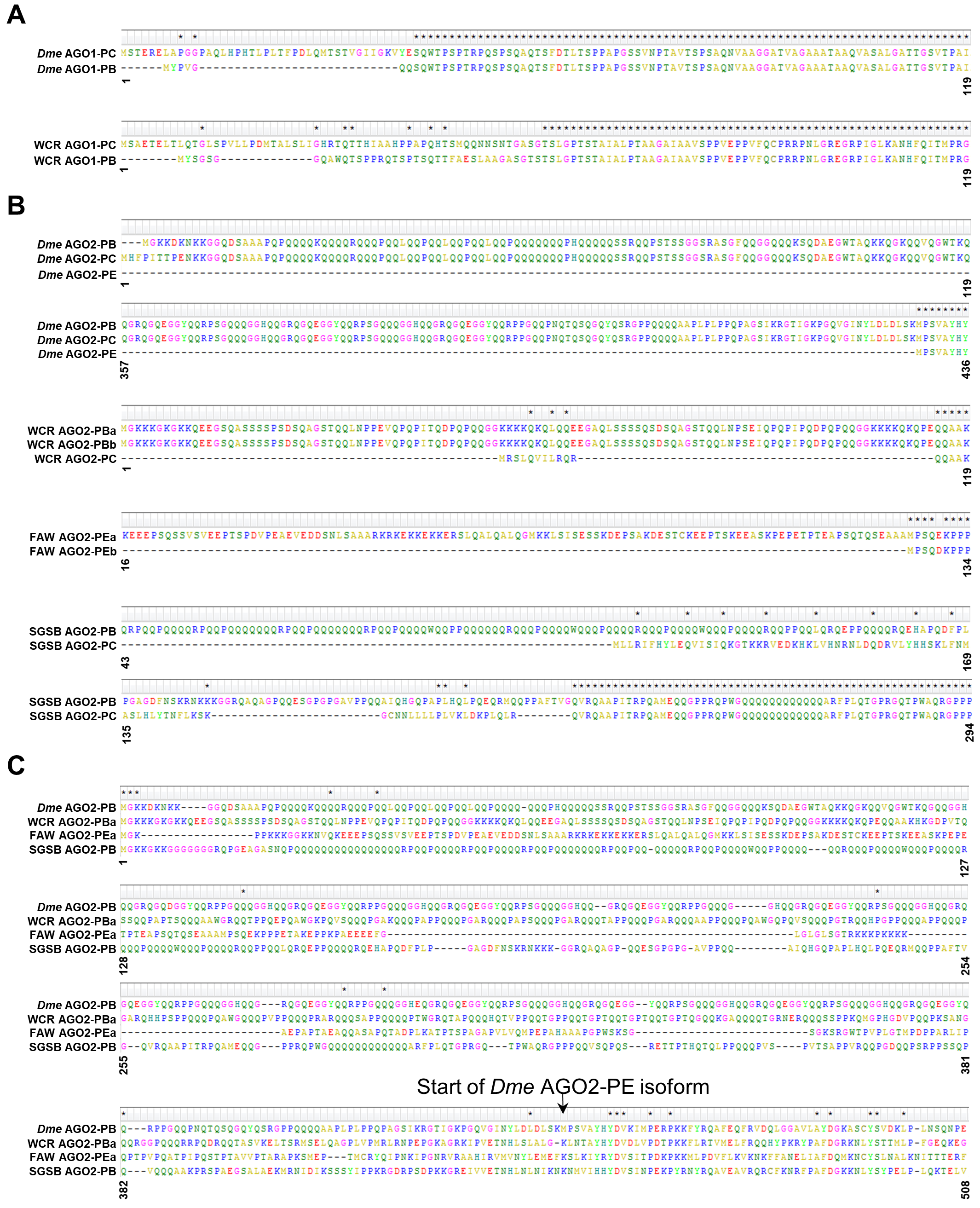

Supplement: S4 Fig — Alignments were performed using ClustalW with default MEGA7 parameters. Analyses for A) and B) were separated by insect to more clearly depict intraspecific sequence differences, which are not easy to visualize in an aggregated alignment. Alignment text colors represent biochemical properties of the different amino acids, and include the following: yellow (A, M, F, I, V, L), olive (C), green (N, Q, S, T, W), aqua (D, E), blue (P), red (R, K), fuchsia (G), teal (H), and lime (Y). Asterisks (*) above the alignment indicate identical residues, and alignment site numbers are shown at the beginning and end of each block. A) Differences at the C-termini of Dme and WCR AGO1 isoforms. B) Differences at the C-termini of Dme, WCR, FAW, and SGSB AGO2 isoforms. C) Alignment of AGO2-PB sequences from Dme, WCR, and SGSB, and AGO2-PEa of FAW. (TIF) [file pone.0203160.s010.tif]
